# Probabilistic quality assurance of biopsy DVH thresholds in HDR prostate brachytherapy under localization uncertainty

**DOI:** 10.1002/acm2.70660

**Published:** 2026-07-19

**Authors:** Matthew Muscat, Juanita Crook, Andrew Jirasek, Jeffrey Andrews, Nathan Becker

**Affiliations:** ^1^ Department of Computer Science, Mathematics, Physics and Statistics University of British Columbia Okanagan Kelowna British Columbia Canada; ^2^ BC Cancer Sindi Ahluwalia Hawkins Centre Kelowna British Columbia Canada; ^3^ Department of Surgery University of British Columbia Kelowna British Columbia Canada

**Keywords:** biopsy dosimetry, dose volume histogram, HDR prostate brachytherapy, localization uncertainty, Monte Carlo simulation, prostate cancer, quality assurance

## Abstract

**Background:**

MR‐US guided biopsies support biologic correlation in HDR prostate brachytherapy, but steep dose gradients and millimeter‐scale localization uncertainty limit the validity of nominal biopsy doses.

**Purpose:**

To assess how localization uncertainty affects the robustness of biopsy DVH dose thresholds in HDR prostate brachytherapy and to quantify the nominal dose margins corresponding to 95% pass probabilities.

**Methods:**

Twenty‐seven biopsies from 15 HDR monotherapy patients were reconstructed as sets of 1mm voxels and sampled on the clinical HDR dose lattice. Localization uncertainty was modeled with 10 000 Monte Carlo trials per biopsy using isotropic 3D Gaussian translations (σ=1.25mm) plus an axial tissue‐deficit offset. For each voxel we computed nominal and Monte Carlo summaries of dose and dose gradient magnitude; for each biopsy we evaluated DVH metrics $D_{2\%}$, $D_{50\%}$, $D_{98\%}$, and $V_{150\%}$. For four robustness thresholds, Monte Carlo pass probabilities $p_{b}$ were related to nominal distance‐from‐threshold using logistic models, and geometric, spatial, and radiomics predictors were tested as secondary covariates.

**Results:**

The pooled voxel‐dose and dose‐gradient distributions were broad, positively skewed, and consistent with log‐normal models. Nominal voxel‐level doses tended to be higher than localization‐perturbed samples, with mean absolute per‐trial $|\Delta D_{b,v,t}|\approx 7.6\;\mathrm{Gy}$. Margin‐only logistic models captured most of the variability in pass probabilities; the nominal margins required for a 95% pass probability were ≈6Gy ($D_{98\%}$), 21Gy ($D_{50\%}$), 49Gy ($D_{2\%}$), and 49 percentage points ($V_{150\%}$). Adding geometric, spatial, or radiomics predictors altered these margins only modestly. Along biopsy cores, mean absolute dose differences between voxel pairs approached ≈10Gy at separations of 1–1.5cm, indicating decorrelation on that scale.

**Conclusions:**

Localization uncertainty causes nominal biopsy DVH metrics to systematically overestimate Monte Carlo‐propagated doses. The reported 95% margins should be interpreted as biopsy‐scale robustness buffers under the stated uncertainty model, not as clinically prescriptive focal‐boost increments. Dose decorrelated over distances of order 1–1.5cm along biopsy cores, supporting spatially explicit, Monte Carlo‐based reporting when interpreting biopsy dosimetry in HDR prostate brachytherapy.

## 1 INTRODUCTION

Magnetic resonance imaging (MRI) is now central to localizing clinically significant prostate cancer and guiding targeted biopsy workflows. Standardized interpretation and reporting through PI‐RADS have supported consistent lesion characterization on multiparametric MRI (mpMRI) and established mpMRI as a practical decision‐making tool in diagnostic pathways.^1^ Randomized evidence has shown that an MRI‐directed pathway with targeted biopsy increases detection of clinically significant disease while simultaneously reducing detection of clinically insignificant disease compared with standard systematic transrectal ultrasound (TRUS) biopsy.^2^ MRI‐US fusion platforms provide one practical implementation of this pathway by bringing MRI‐defined targets into the intraoperative ultrasound domain, and have been associated with higher detection of higher‐risk cancer and lower detection of low‐risk cancer than systematic biopsy alone.^3^ Biopsy Gleason Grade Group and tumor volume estimates feed directly into risk stratification and treatment selection, so both targeted and targeted‐plus‐systematic strategies are used to improve grading concordance with final pathology.^4^ In all of these settings, however, MRI‐US fusion guidance does not make biopsy localization exact: millimeter‐scale uncertainty from registration, segmentation, needle placement, and gland deformation means that the effective sampling site can be displaced from the intended target.

In high‐dose‐rate (HDR) prostate brachytherapy, the same imaging paradigm is directly relevant because the technique delivers highly conformal dose distributions with steep spatial gradients.^5^ Contemporary practice guidelines emphasize quantitative dose reporting and plan evaluation using dose‐volume histogram (DVH) metrics and DVH‐based constraints for targets and organs at risk.^6^, ^7^ Clinical series have reported excellent target coverage with HDR brachytherapy, yet local recurrences frequently arise in or near the dominant intraprostatic lesion (DIL) or site of initial disease identified on baseline biopsies or mpMRI.^8^, ^9^, ^10^, ^11^, ^12^, ^13^ These patterns suggest that, beyond global target coverage, biopsy‐scale spatial dose information may be important for understanding local control and for interpreting biopsy‐based biological assays.

Prostate cancer exhibits marked intra‐ and inter‐tumour genomic and phenotypic heterogeneity, with distinct clonal subpopulations and spatially variable molecular profiles that can influence radiosensitivity and patterns of progression.^14^, ^15^, ^16^, ^17^, ^18^, ^19^ The tumour microenvironment, including hypoxia and related signaling pathways, has also been implicated in radioresistance and treatment failure in prostate cancer.^20^, ^21^, ^22^, ^23^, ^24^ These considerations have motivated biologically informed risk stratification using genomic signatures and other tissue‐based assays,^25^, ^26^, ^27^ and recent work has demonstrated the feasibility of Raman spectroscopy to probe biochemical composition and treatment response in prostate tissue, including HDR brachytherapy cohorts.^28^, ^29^ In this context, MR‐US guided biopsies provide a natural means of linking localized tissue measurements to spatially resolved dose information.

For biopsy‐dose correlation studies, the standard HDR brachytherapy framework provides a convenient clinical vocabulary: treating a core as a small sampled volume allows biopsy dosimetry to be summarized with DVH‐style coverage and hotspot metrics that align with routine HDR plan evaluation. However, the steep gradients that enable conformal HDR dose distributions also amplify localization uncertainty. Millimeter‐scale uncertainties in needle placement, image registration, segmentation, and anatomical deformation can translate into clinically meaningful uncertainty in the dose assigned to a small tissue volume, and medical physics guidance has stressed the importance of validating and reporting image registration performance for this reason.^30^ Reporting a single nominal dose or DVH metric for a biopsy therefore obscures both the spread of plausible dose values and the likelihood that the biopsy satisfies a clinically meaningful dose threshold, or a threshold used in biological correlation studies.

Previous work developed a voxel‐level framework for propagating localization uncertainty onto MR‐informed, ultrasound‐guided prostate biopsy cores in HDR brachytherapy.^31^ Biopsies were reconstructed as sets of 1 mm voxels, sampled on the clinical HDR dose lattice, and subjected to a Monte Carlo localization‐uncertainty model comprising isotropic 3D Gaussian translations plus an axial tissue‐deficit offset. That study focused on a small number of exemplar cores and showed that nominal voxel and biopsy assignments can differ from localization‐perturbed realizations by several Gy, with centimeter‐scale decorrelation of dose along individual cores. In a companion paper, the same localization‐uncertainty framework was used to construct a probabilistic tissue‐classification metric for MR‐US guided biopsies. Monte Carlo propagated sampling locations, tested against the MRI‐derived structure set, were combined to model the probability that a biopsy samples a given tissue class, and a framework was developed to validate this model against histopathology.^32^ Together, these works implemented and applied the localization‐uncertainty model and demonstrated its impact on voxel‐level dose assignment and biopsy‐level tissue classification, but they did not assess propagated dosimetric uncertainty at the cohort level, nor provide a cohort‐level quality assurance assessment of DVH‐style biopsy thresholds under uncertainty.

The purpose of this work is to extend the biopsy‐localization uncertainty model from exemplar cores to cohort‐level DVH‐style quality assurance of MR‐US guided prostate biopsies in HDR brachytherapy. Using 27 biopsies from 15 HDR monotherapy patients, we aim to: (i) summarize pooled voxel‐level and biopsy‐level dose and dose‐gradient distributions and quantify discrepancies between nominal and Monte Carlo‐propagated values using voxel‐wise and biopsy‐level delta metrics; and (ii) evaluate biopsy DVH criteria based on $D_{2\%}$, $D_{50\%}$, $D_{98\%}$, and $V_{150\%}$ by translating them into Monte Carlo pass probabilities and confidence classes under the fixed localization‐uncertainty model. We then relate robustness to nominal dosimetric distance‐from‐threshold, assess the incremental contribution of geometric, spatial, and radiomics‐style predictors, and characterize intra‐core decorrelation length scales for dose and dose gradient.

## 2 MATERIALS AND METHODS

**FIGURE 1:**
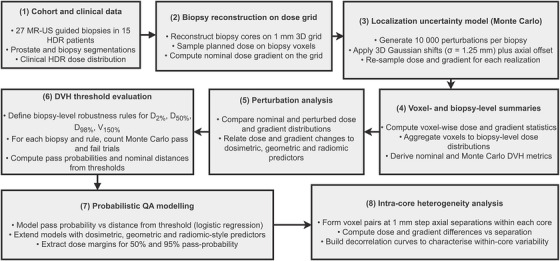
Schematic of the biopsy‐level Monte Carlo DVH QA pipeline, from cohort selection and biopsy reconstruction through localization‐uncertainty modeling, voxel‐ and biopsy‐level dose summaries, perturbation analysis, DVH threshold evaluation, probabilistic QA modeling, and intra‐core heterogeneity analysis.

Rigorous definitions of all random variables, operators, and pooling constructions used in this work are provided in Appendix B. In this section we summarise the core elements of the framework and reference the corresponding equations as needed. All biopsy localization modeling steps were implemented in Python using an open‐source biopsy localization pipeline.^33^ Dose accumulation and DVH analyses were performed using a dedicated dosimetry and statistical analysis pipeline.^34^ The overall analysis workflow is summarized in Figure 1.

### 2.1 Clinical dataset and analysis scope

This study repurposes clinical data (Table 1) from patients enrolled on a prospective trial comparing low‐dose‐rate and high‐dose‐rate (HDR) prostate brachytherapy (H17‐02904, H24‐00948 and H23‐02872). The present analysis includes only biopsies acquired during the second HDR implantation procedure, after the first HDR fraction and before delivery of the second fraction, so that each analyzed core corresponds to tissue previously exposed to one delivered fraction (typically about two weeks earlier). Patients in the HDR monotherapy arm received 27Gy in two fractions of 13.5Gy to the clinical target volume (CTV). Planning and guidance imaging included multiparametric MRI (mpMRI, 1.5T, endorectal coil) and intraoperative transrectal ultrasound (TRUS). Dominant intraprostatic lesions (DILs) were identified on the target‐definition mpMRI sequences (T2‐weighted, diffusion‐weighted, and dynamic contrast‐enhanced imaging). The T2‐weighted and diffusion‐weighted/ADC sequences used for lesion delineation had 1mm slice thickness. DILs were transferred to a pre‐brachytherapy TRUS dataset using rigid MRI‐TRUS registration with the prostate and urethra as anatomical landmarks. Biopsies were then cognitively targeted under live TRUS guidance. Rigid MRI‐TRUS registration and target transfer for this workflow were performed using MIM (MIM Software Inc., Beachwood, USA) and Vitesse (Varian Medical Systems Inc., Palo Alto, USA).

**TABLE 1:** Summary of the patient cohort, HDR brachytherapy protocol, and biopsies included in the localization‐uncertainty and Monte Carlo DVH QA analysis (H17‐02904, H24‐00948 and H23‐02872).

Characteristic	Value
Study arm	HDR brachytherapy monotherapy
Risk group	Intermediate‐risk prostate cancer
Patients	15
Gleason score	3 + 3, 3 + 4, or 4 + 3
Dose prescription	27 Gy in 2 × 13.5 Gy fractions
Imaging (target definition)	mpMRI with endorectal coil
Intraoperative guidance	TRUS with MRI‐US fusion
Biopsies analyzed	27 cores
Targeted DILs	26 mpMRI‐defined lesions

Table 1 summarises the cohort and protocol. In total, 27 MR‐US guided biopsy cores from 15 HDR monotherapy patients were available for analysis, targeting 26 mpMRI‐defined dominant intraprostatic lesions (DILs).

### 2.2 Biopsy reconstruction and voxel‐level dosimetry

Each MR‐US guided biopsy core b was reconstructed as a connected set of cubic voxels on a regular 1mm grid along the recorded core axis. Voxels were indexed in order along the axis $v=1,\cdots ,n_{b}$, with a corresponding axial coordinate $z_{b,v}$ and spacing Δz=1mm. The 3D center of voxel v in the nominal (unperturbed) configuration is denoted ${\vec{x}}_{b,v}\left(0\right)$.

Clinical HDR dose, sampled from the planning lattice exported from Vitesse and reflecting the standard TG‐43‐based calculation in a homogeneous water medium used clinically, was available on a rectilinear lattice $D(\vec{x})$, typically at $1\times 1\times 0.5\;{\mathrm{mm}}^{3}$ spacing. The norm of the local dose gradient, $∥\nabla D∥\left(\vec{x}\right)\equiv ∥\nabla D\left(\vec{x}\right)∥$, was computed on this lattice, so that ∥∇D∥ has units of $\text{Gy}\;{\text{mm}}^{-1}$ (Equation (B1)).

Voxel‐level dose and gradient for the biopsies were obtained by interpolating the background dose and gradient lattices at the biopsy voxel centers. We used four‐nearest‐neighbor inverse‐distance‐weighted interpolation with a coincident‐point convention for both $D(\vec{x})$ and $∥\nabla D∥(\vec{x})$ (Equation (B2)). For brevity we denote the interpolated dose and gradient at voxel v in biopsy b and Monte Carlo trial t by $D_{b,v}^{\left(t\right)}$ and $G_{b,v}^{\left(t\right)}$, respectively. The nominal (unperturbed) quantities correspond to t=0.

### 2.3 Localization‐uncertainty Monte Carlo model

Localization uncertainty for each biopsy core was modeled as a rigid 3D translation applied to the entire voxelized biopsy, together with an axial offset to account for potential tissue deficit within the biopsy needle.

We performed $N_{T}=10,000$ localization‐perturbation trials per biopsy in addition to the nominal configuration. Trials were indexed by $t=0,1,\cdots ,N_{T}$, with t=0 denoting the nominal case and t≥1 denoting localization‐perturbation trials (Equation (B3)).

For each perturbed trial t≥1, a translation vector ${\vec{T}}_{t}$ was sampled from an isotropic 3D normal distribution with zero mean and standard deviation σ=1.25mm in each direction (Equation (B4)). This choice of σ follows previous probabilistic biopsy‐localization work,^32^ where it was introduced as a first‐order, millimeter‐scale approximation that aggregates dominant sources of targeting and registration uncertainty; detailed justification and supporting discussion are provided there. The translation ${\vec{T}}_{t}$ was applied uniformly to all voxels in the biopsy, preserving biopsy connectivity under a rigid‐motion prior.

To model possible mismatch between the biopsy needle compartment length and the extracted tissue length, an independent axial tissue‐deficit offset was drawn along the core axis for each trial. Specifically, if $l_{c}$ denotes the needle compartment length and $l_{a}$ the extracted tissue length, then the axial offset was sampled as $X_{l,t}∼U\left(0,\max\left(l_{c}-l_{a},0\right)\right)$ and combined with ${\vec{T}}_{t}$ to give the total trial‐specific translation ${\vec{T}}_{t}$ (Equation (B5)).

The translated voxel centers are thus ${\vec{x}}_{b,v}\left(t\right)={\vec{x}}_{b,v}\left(0\right)+{\vec{T}}_{t}$, and the corresponding sampled dose and gradient are $D_{b,v}^{\left(t\right)}$ and $G_{b,v}^{\left(t\right)}$ (Equations (B6) to (B7)).

For each biopsy, voxel, and trial we then sampled the interpolated dose and dose‐gradient magnitude from the background lattices at ${\vec{x}}_{b,v}\left(t\right)$, yielding trialwise sets ${\left\{D_{b,v}^{\left(t\right)}\right\}}_{t=0}^{N_{T}}$ and ${\left\{G_{b,v}^{\left(t\right)}\right\}}_{t=0}^{N_{T}}$. The full mathematical formulation of the localization model is given in Appendix B.

### 2.4 Voxel‐, biopsy‐, and cohort‐level summaries

Analyses were performed at three linked levels (voxel, biopsy, and cohort), delaying averaging so that localization uncertainty could be examined at the scale of individual voxels, whole cores, and the pooled cohort. The full mathematical formulation of the pooling operations is given in Section B.3.

#### Voxel level

For each biopsy voxel (b,v), localization uncertainty generated trialwise dose and gradient samples ${\left\{D_{b,v}^{\left(t\right)},G_{b,v}^{\left(t\right)}\right\}}_{t=1}^{N_{T}}$, as defined in Equation (B8). From these Monte Carlo sets we computed voxel‐level summaries (mean, median, selected quantiles, standard deviation, and a kernel‐density‐estimate mode*) and compared them with the nominal voxel values $D_{b,v}^{\left(0\right)}$ and $G_{b,v}^{\left(0\right)}$. These quantities provide uncertainty‐propagated dose and dose‐gradient estimates at each voxel.

#### Biopsy level

Within each biopsy, voxelwise trial samples were pooled across voxels to form biopsy‐level dose and gradient distributions $D_{b},G_{b}$ (Equation (B9)). From these we obtained biopsy‐level central values (for example, median dose) and uncertainty widths (for example, central 90% interval width, IPR90). Nominal biopsy‐level dose and gradient were defined as the core‐averaged values ${\bar{D}}_{b}^{\left(0\right)}$ and ${\bar{G}}_{b}^{\left(0\right)}$, obtained by averaging $D_{b,v}^{\left(0\right)}$ and $G_{b,v}^{\left(0\right)}$ over voxels (Equation (B10)).

#### Cohort level

Cohort‐level pooled distributions were formed by concatenating biopsy‐level collections across all B=27 biopsies (Equation (B11)). In parallel, biopsy‐level summaries (such as ${\bar{D}}_{b}^{\left(0\right)}$ and biopsy‐level medians) were aggregated across biopsies to characterize inter‐biopsy heterogeneity.

Cohort‐pooled voxel‐level values of dose and dose gradient were also summarized with standard parametric families, fit by maximum likelihood and compared using Akaike's information criterion (AIC). For the metrics used in this work, three‐parameter log‐normal models provided the best AIC in all cases. The candidate families, fitting procedure, and log‐normal parameterization are detailed in Appendix B.4.

### 2.5 Nominal versus Monte Carlo‐propagated delta metrics

To quantify differences between nominal voxel assignments and uncertainty‐propagated summaries, we defined a family of delta metrics.

#### Voxelwise summary deltas

For each voxel we compared the nominal dose $D_{b,v}^{\left(0\right)}$ to Monte Carlo propagated summaries of the perturbed trials. Let $D_{b,v}^{\left(\mathrm{mode}\right)}$, $D_{b,v}^{\left(Q50\right)}$, and $D_{b,v}^{\left(\mathrm{mean}\right)}$ denote the dose mode, median, and mean computed from the perturbed trials at voxel v. The corresponding deltas $\Delta D_{b,v}^{\left(j\right)}$ were defined as nominal minus Monte Carlo summary, with j∈{mode,Q50,mean} (Equation (B17)). Analogous definitions were used for the dose‐gradient magnitude, yielding $\Delta G_{b,v}^{\left(j\right)}$. We report cohort‐level summaries of the absolute deltas $|\Delta D_{b,v}^{\left(j\right)}|$ and $|\Delta G_{b,v}^{\left(j\right)}|$, pooled over voxels and biopsies.

#### Per‐trial deltas

At the per‐trial level we compared the nominal voxel value to each perturbed realization, defining $\Delta D_{b,v,t}=D_{b,v}^{\left(0\right)}-D_{b,v}^{\left(t\right)}$ and similarly for the dose gradient (Equation (B19)). These per‐trial deltas were also pooled across voxels, trials, and biopsies to characterize the typical absolute difference between the nominal assignment and a single perturbed draw.

#### Directionality

To summarize whether nominal values tend to over‐ or under‐estimate uncertainty‐propagated draws, we used the common‐language effect size (CLES), that is, the probability that a randomly chosen delta is positive plus half the probability that it is zero. The formal definition of the CLES is given in Equation (B21).

#### Voxel‐level correlations with candidate predictors

To relate voxel‐level discrepancies to local anatomy and dosimetry, we computed Pearson correlations between absolute voxel‐level deltas, $|\Delta D_{b,v}^{\left(j\right)}|$, and a set of dosimetric, geometric, and radiomics‐style predictors (Appendix A), pooling voxels across biopsies. These correlations were used descriptively to rank predictors and to select representative examples for voxel‐level scatterplots in Section 3.4.3.

### 2.6 DVH‐style biopsy metrics and thresholds

For each biopsy and each Monte Carlo trial we computed DVH‐style biopsy metrics from the trial‐specific voxel doses ${\left\{D_{b,v}^{\left(t\right)}\right\}}_{v=1}^{n_{b}}$. For a given percentage x∈(0,100), the biopsy‐level dose metric $D_{b,x\%}\left(t\right)$ was defined as the minimum dose to the hottest x% of biopsy voxels, that is, the (1−x/100) quantile of the voxel‐dose distribution within biopsy b for trial t, as formalized in Equation (B22).

For prescription‐referenced volume metrics we took the per‐fraction prescription $D_{\mathrm{Rx}}=13.5\;\mathrm{Gy}$. For x∈(0,300), the biopsy‐level volume metric $V_{b,x\%}\left(t\right)$ was defined as the fraction of biopsy voxels receiving at least $\left(x/100\right)D_{\mathrm{Rx}}$, as in Equation (B23).

In this work we describe cohort‐level distributions for $D_{2\%}$, $D_{50\%}$, $D_{98\%}$ and the hotspot‐style volume metrics $V_{100\%}$, $V_{125\%}$, $V_{150\%}$, $V_{175\%}$, $V_{200\%}$, and $V_{300\%}$. The quality‐assurance analysis focuses on four DVH robustness rules constructed from $D_{2\%}$, $D_{50\%}$, $D_{98\%}$, and $V_{150\%}$.

For brevity we write $M_{b,r}\left(t\right)$ for the DVH metric associated with rule r in trial t and $T_{r}$ for its corresponding threshold. The four rules are

(1) $$\begin{array}{cc} r=1 & :\;D_{b,2\%}\left(t\right)\geq 32\;\text{Gy},\\ r=2 & :\;D_{b,50\%}\left(t\right)\geq 27\;\text{Gy},\\ r=3 & :\;D_{b,98\%}\left(t\right)\geq 20\;\text{Gy},\\ r=4 & :\;V_{b,150\%}\left(t\right)\geq 50\%. \end{array}$$

These four rules were selected as illustrative biopsy‐scale QA examples spanning coverage‐ and hotspot‐type behavior in this cohort; they are used to demonstrate the framework rather than to define definitive clinical planning constraints.

The indicator for rule r in trial t is denoted $I_{b,r}^{\left(t\right)}=I\left[M_{b,r}\left(t\right)\geq T_{r}\right]$, which is equal to 1 if the threshold is satisfied and 0 otherwise. The associated pass probabilities are described in Section 2.7 and defined in Equation (B26).

### 2.7 Quality assurance of DVH‐style biopsy criteria under localization uncertainty

For each biopsy b, rule r, and perturbed trial t≥1, the DVH rule was evaluated using the indicator $I_{b,r}^{\left(t\right)}$ defined in Equation (B25). Threshold robustness under the localization‐uncertainty model was summarized by the Monte Carlo pass probability $p_{b,r}$ (fraction of perturbed trials that pass), as defined in Equation (B26). Biopsy‐rule pairs were classified as confident pass ($p_{b,r}\geq 0.95$), confident fail ($p_{b,r}\leq 0.05$), or borderline ($0.05<p_{b,r}<0.95$).

The nominal distance from threshold (“margin”) is $\delta_{b,r}\equiv M_{b,r}\left(0\right)-T_{r}$ (Equation (B27)), so that $\delta_{b,r}>0$ corresponds to a nominal pass and $\delta_{b,r}<0$ to a nominal fail. For dose‐based rules $\delta_{b,r}$ is measured in Gy; for the volume‐based rule it is measured in percentage points.

#### Margin‐only logistic model

To relate threshold robustness to nominal margin, we modeled the number of passing perturbed trials $n_{\mathrm{pass},b,r}$ (Equation (B28)) as a binomial count with underlying pass probability $\pi_{b,r}$, linked to $\delta_{b,r}$ by a single‐predictor logistic regression,

(2) $$\mathrm{logit}\left(\pi_{b,r}\right)=\beta_{0,r}+\beta_{1,r}\;\delta_{b,r},$$

fit separately for each rule r (cf. Equation (B30)). For each rule we report the nominal margins ${\hat{\delta }}_{0.50,r}$ and ${\hat{\delta }}_{0.95,r}$ at which the fitted model predicts $\pi_{b,r}=0.5$ and $\pi_{b,r}=0.95$, respectively.

#### Secondary predictors

In a secondary analysis, we extended the model to include a single biopsy‐level predictor $g_{b,r}$ (for example, nominal core‐mean gradient, distances to surrounding structures, or radiomics‐style DIL shape features; Appendix A). The extended models have the standard two‐predictor logit form (Equation (B31)). For each rule r and each candidate $g_{b,r}$, we compared the margin‐only and margin‐plus‐predictor fits using ΔAIC and nested likelihood‐ratio tests, and denoted the best secondary predictor by ${\tilde{g}}_{r}$. Its effect on robustness was summarized by the change in required 95%‐pass margin, $\Delta {\hat{\delta }}_{0.95}$, and the scale‐free ratio $\Delta {\hat{\delta }}_{0.95}/\sigma_{r}$, defined in Equations (B32) and (B33) and reported in Table C13.

#### Influential outlier for the $D_{2\%}$ rule

For the $D_{2\%}\geq 32\;\text{Gy}$ threshold (rule r=1), one biopsy was excluded from the analysis after Cook's distance diagnostics identified it as an influential outlier.† All reported $D_{2\%}$ summaries are therefore based on N=26 biopsies.

### 2.8 Intra‐core spatial heterogeneity from voxel‐pair differences

To characterize how rapidly dose and dose gradient can vary along a biopsy, we analyzed voxel‐pair differences within each core.

#### Voxel‐index heatmaps

For each biopsy b, voxel indices i,j, and trial t, we computed pairwise differences in dose and gradient, $D_{b,i}^{\left(t\right)}-D_{b,j}^{\left(t\right)}$ and $G_{b,i}^{\left(t\right)}-G_{b,j}^{\left(t\right)}$. These were then converted to absolute differences and pooled across all trials and all biopsies that were sufficiently long to contribute a given index pair (i,j). Mean absolute differences were displayed in a heatmap, with the upper triangle encoding dose and the lower triangle encoding dose‐gradient magnitude. Near‐diagonal cells represent short separations along the core, whereas far‐off‐diagonal cells represent long separations.

#### Length‐scale dependence

To summarize voxel‐pair differences as a function of axial separation, we grouped voxel pairs by the separation $\ell_{k}=k\Delta z$ between their indices, where Δz=1mm and k≥1. For each separation we pooled absolute dose and gradient differences across biopsies and trials, obtaining cohort‐level distributions and summary statistics (mean, median, interquartile range, and tail percentiles) as functions of $\ell_{k}$. The number of contributing voxel pairs naturally decreases at large separations because fewer cores are long enough to support them. Formal definitions of the voxel‐pair matrices and length‐scale sets are provided in Equations (B34) to (B36).

## 3 RESULTS

### 3.1 Segmentation context and radiomic features

Radiomic features for the prostate and dominant intraprostatic lesions (DILs) have been described previously for this cohort,^31^ and are summarized here to provide anatomical context for the dosimetric analyses. Individual DIL volumes had a mean ± SD of $1.4\pm 1.0\;{\mathrm{cm}}^{3}$, with cumulative DIL volume per patient of $2.5\pm 1.6\;{\mathrm{cm}}^{3}$, compared with a mean prostate volume of $44.3\pm 10.5\;{\mathrm{cm}}^{3}$. Thus, individual targets were small relative to the gland, and biopsies sampled tissue within a heterogeneous background of lesion shapes and prostate sizes. Biopsy core lengths had a mean ± SD of 15.5±2.1mm, and the baseline centroid‐to‐centroid distance between each biopsy and its targeted DIL had a mean ± SD of 7.2±3.4mm.

### 3.2 Cohort‐level voxel‐wise dose and dose‐gradient distributions

**FIGURE 2:**
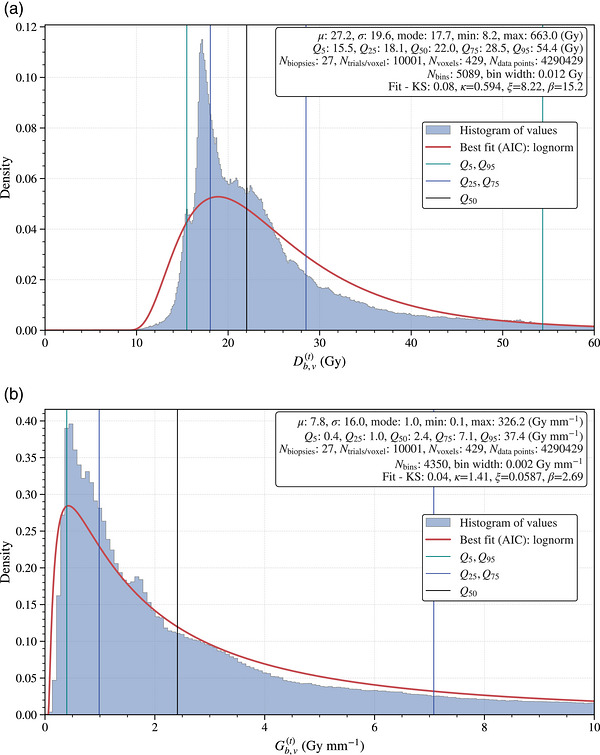
Cohort‐level distributions of voxel‐wise dose and dose‐gradient magnitude pooled over all biopsy voxels and uncertainty trials (27 biopsies in 15 patients). (A) Distribution of voxel‐wise dose $D_{b,v}^{\left(t\right)}$ (Gy). (B) Distribution of voxel‐wise dose‐gradient magnitude $G_{b,v}^{\left(t\right)}$ ($\text{Gy}\;{\text{mm}}^{-1}$). For each quantity, candidate parametric distributions were fitted and compared using the Akaike information criterion; in both cases, a log‐normal model was preferred. Fitted parameters and goodness‐of‐fit statistics are reported in the figure legends, using the standard log‐normal parameterization described in Methods section 2.4 and Appendix B.4. Histogram bin counts were chosen using a bounded Freedman‐Diaconis rule (IQR‐based).

The pooled voxel‐wise dose distribution across all biopsies and Monte Carlo trials was broad and positively skewed (Figure 2A). The mean ± SD sampled dose was 27.2±19.6Gy, with median 22.0Gy and an upper tail extending beyond 50Gy (95th percentile 54.4Gy; Table C1). Model selection over the candidate distribution family identified a log‐normal model as the best by AIC, consistent with heavy right‐tailed behavior.

The corresponding cohort‐level distribution of dose‐gradient magnitude $G_{b,v}^{\left(t\right)}$ showed a similar pattern (Figure 2B). The mean ± SD gradient was $7.8\pm 16.0\;\text{Gy}\;{\text{mm}}^{-1}$, with median $2.4\;\text{Gy}\;{\text{mm}}^{-1}$ and a long upper tail (95th percentile $37.4\;\text{Gy}\;{\text{mm}}^{-1}$; Table C1). A log‐normal model again provided the best fit by AIC. Together, these distributions confirm that biopsy voxels sample both low‐gradient plateau regions and very steep gradients near dwell positions, and that cohort‐level interpretation must accommodate substantial skew and heavy tails in both dose and gradient.

### 3.3 Biopsy‐ and voxel‐level dosimetry and DVH metrics

Here and in relevant tables, values reported as x±y denote the cohort mean and standard deviation of the quantity in question (across biopsies, voxels, or patients, as specified) unless otherwise stated.

Across pooled localization trials, rigid per‐axis shifts remained centred near zero (|μ|<0.004mm) with empirical standard deviations of ≈1.26mm and 5th/95th percentiles of approximately ±2.07mm, while the axial tissue‐deficit offset had mean 1.80mm, median 1.39mm, 5th/95th percentiles 0.03mm and 4.83mm, and maximum 7.48mm.

#### 3.3.1 Biopsy‐ and voxel‐level dose and gradient summaries

At the biopsy level, nominal and Monte Carlo‐propagated summaries indicated that most cores received doses well above the per‐fraction prescription, with substantial variability between biopsies. Across the N=27 biopsies, the nominal core‐averaged dose ${\bar{D}}_{b}^{\left(0\right)}$ was 29.0±15.2Gy, whereas the biopsy‐level Monte Carlo median dose (the median of the pooled set $D_{b}$) was 24.3±7.5Gy (Table C3). The central 90% interval width (IPR90) of the biopsy‐level Monte Carlo dose distribution under localization perturbations was 31.8±32.0Gy, indicating that some cores exhibited very wide trial‐to‐trial ranges while others were relatively stable.

For the dose gradient, the nominal core‐averaged gradient ${\bar{G}}_{b}^{\left(0\right)}$ was $11.5\pm 19.0\;\text{Gy}\;{\text{mm}}^{-1}$, compared with a biopsy‐level Monte Carlo median gradient of $4.7\pm 6.1\;\text{Gy}\;{\text{mm}}^{-1}$ and an IPR90 of $28.1\pm 34.4\;\text{Gy}\;{\text{mm}}^{-1}$ (Table C3).

A similar pattern held at the voxel level. Across the cohort of $N_{\text{voxels}}=429$ sampled biopsy voxels, the nominal assigned voxel‐level dose $D_{b,v}^{\left(0\right)}$ was 28.5±18.8Gy. The voxel‐level Monte Carlo mean dose (mean over trials for each voxel) was 27.2±11.6Gy, and the mean gradient was $7.8\pm 10.1\;\text{Gy}\;{\text{mm}}^{-1}$, consistent with the cohort histograms and emphasising that voxel‐level exposure remains heterogeneous even after averaging over trials (Table C2).

#### 3.3.2 DVH‐style biopsy metrics

**FIGURE 3:**
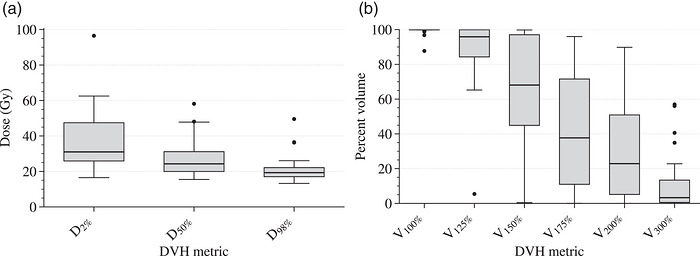
Box‐plots of per‐biopsy DVH metrics. (A) Dose‐based $D_{x\%}$ metrics. (B) Volume‐based $V_{x\%}$ metrics. Each box‐plot summarizes 27 biopsies across 15 patients, where each biopsy's value is the mean of the corresponding metric across all uncertainty trials. The $D_{x\%}$ metrics are the minimum dose received by the highest x% of voxel doses, and the $V_{x\%}$ metrics are the percentage of biopsy volume that received at least x% of the prescription dose (13.5Gy).

DVH‐style coverage and hotspot metrics for each biopsy are summarized in Figure 3 and Table C4. For the dose metrics, the cohort mean ± SD of the nominal biopsy‐level $D_{2\%}$, $D_{50\%}$, and $D_{98\%}$ were 41.6±39.7Gy, 28.2±14.3Gy, and 22.1±10.0Gy, respectively. The corresponding Monte Carlo median summaries were 29.7±9.7Gy for $D_{2\%}$, 24.8±8.0Gy for $D_{50\%}$, and 20.4±5.8Gy for $D_{98\%}$, with the widest IPR90 widths observed for $D_{2\%}$ (mean 61.8±78.4Gy) and narrower intervals for $D_{50\%}$ and $D_{98\%}$. Overall, most biopsies receive coverage consistent with or above prescription in central voxels, while the highest‐dose portions of some cores show very wide Monte Carlo ranges.

For volume‐based metrics referenced to the per‐fraction prescription $D_{\mathrm{Rx}}=13.5\;\mathrm{Gy}$, nominal $V_{100\%}$ values were tightly clustered near full coverage (mean 99.8±1.1%, Monte Carlo median 99.6±2.3%). In contrast, the hotspot metric $V_{150\%}$ showed much greater variability, with nominal 66.1±38.3%, Monte Carlo median 63.0±37.8%, and a mean IPR90 of 39.1±32.3 percentage points. Higher‐dose volumes ($V_{175\%}$, $V_{200\%}$, $V_{300\%}$) spanned almost the full 0‐100% range across biopsies (Table C4), indicating that some cores lay almost entirely within high‐dose regions while others only partially intersected hotspots.

### 3.4 Nominal versus Monte Carlo propagated dose assignments

#### 3.4.1 Voxel‐level summary deltas

Voxel‐level comparisons between nominal values and Monte Carlo summaries showed nontrivial discrepancies, especially for mode‐based estimates and in high‐gradient regions. Cohort‐pooled voxel‐level absolute deltas $|\Delta D_{b,v}^{\left(j\right)}|$ and $|\Delta G_{b,v}^{\left(j\right)}|$ are summarized in Table C5. For dose, the median absolute voxel‐wise delta was 1.2Gy for the mode‐based summary, with a 95th percentile of 28.7Gy. The corresponding medians for the Monte Carlo median and mean summaries were 0.9 and 1.1Gy, with 95th percentiles of 22.2 and 13.3Gy, respectively. For the dose gradient, median absolute deltas were 0.7, 0.6, and $1.1\;\text{Gy}\;{\text{mm}}^{-1}$ for the mode, median, and mean summaries, with 95th percentiles of 44.1, 32.6, and $25.0\;\text{Gy}\;{\text{mm}}^{-1}$, respectively. Full means and standard deviations for these skewed distributions are reported in Table C5.

Extended statistics in Table C5 show that the signed deltas are typically positive for mode and median (nominal exceeding Monte Carlo‐propagated summaries) and closer to zero for the mean, with occasional large positive excursions. Overall, replacing nominal voxel doses with Monte Carlo summaries would shift many voxels by several Gy on average, with some voxels showing much larger corrections. Thus, nominal, mode, median, and mean voxel‐level dose summaries are separated by several Gy on average, and the choice of summary is nontrivial; we return to the implications of these discrepancies in the Discussion (Section 4).

#### 3.4.2 Per‐trial deltas and directionality

When deltas were defined at the per‐trial level by comparing the nominal value to each localization‐perturbed realization, the pooled mean absolute difference $|\Delta D_{b,v,t}|$ was 7.6Gy with a 95th percentile of 33.0Gy, and the corresponding values for $|\Delta G_{b,v,t}|$ were $7.7\;\text{Gy}\;{\text{mm}}^{-1}$ and $37.2\;\text{Gy}\;{\text{mm}}^{-1}$ (Table C6). The central 90% interval widths (IPR90) of the absolute per‐trial deltas were 32.9Gy for dose and $37.2\;\text{Gy}\;{\text{mm}}^{-1}$ for the dose‐gradient magnitude, indicating substantial trial‐to‐trial variability at the voxel scale.

Directionality, summarized by the common‐language effect size (CLES), showed a mild but consistent tendency for nominal values to exceed uncertainty draws. Pooled over all voxel‐trial comparisons, the probability that the nominal dose exceeded a perturbed trial was 0.58 for dose and 0.55 for gradient. Biopsy‐level CLES values had medians of 0.56 (IQR 0.47‐0.66) for dose and 0.55 (IQR 0.48‐0.62) for gradient. These CLES summaries are not tabulated. Thus, the nominal assignment is not an unbiased representative draw from the localization‐perturbed distribution; it tends to overestimate both dose and gradient slightly, with occasional differences that are clinically meaningful at the voxel scale.

#### 3.4.3 Dependence of voxel‐level deltas on dosimetric, geometric, and radiomic predictors

**FIGURE 4:**
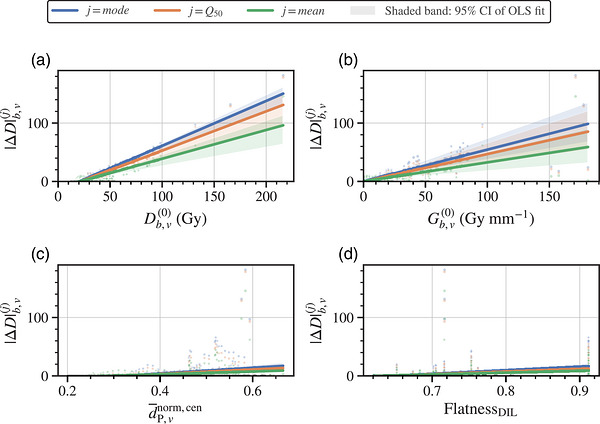
Voxel‐level absolute dose deltas $|\Delta D_{b,v}^{\left(j\right)}|$ versus four representative predictors: nominal dose $D_{b,v}^{\left(0\right)}$ (A), nominal dose gradient $G_{b,v}^{\left(0\right)}$ (B), normalized biopsy‐prostate centroid distance ${\bar{d}}_{P,v}^{\mathrm{norm},\mathrm{cen}}$ (C), and DIL flatness ${\mathrm{Flatness}}_{\mathrm{DIL}}$ (D). Each point corresponds to a single biopsy voxel. In the DIL flatness panel, all voxels from the same biopsy share the same predictor value, which produces vertical bands of points. Across predictors, nominal dose and nominal dose gradient show strong positive associations with $|\Delta D_{b,v}^{\left(j\right)}|$, whereas geometric and radiomic predictors exhibit only weak‐to‐moderate trends, consistent with the modest incremental value of secondary predictors in the logistic models.

Relationships between absolute voxel‐level dose deltas and candidate predictors are summarized in Figure 4. For each summary choice j∈{mode,Q50,mean}, we computed Pearson correlations between $\left|\Delta D_{b,v}^{\left(j\right)}\right|$ and a pool of dosimetric, geometric, and radiomic predictors, and selected four representative predictors with relatively large correlations for visualization. Three of these (nominal voxel dose, nominal voxel dose gradient, and a voxel‐structure distance) are voxel‐level quantities, whereas the fourth (DIL flatness) is defined per biopsy, so all voxels from the same biopsy share the same value and appear as vertical bands. Nominal dose and nominal dose gradient showed strong positive monotonic associations with $\left|\Delta D_{b,v}^{\left(j\right)}\right|$ (Pearson r≈0.89‐0.95 and 0.73‐0.83 across j), while geometric and radiomic predictors exhibited only weak‐to‐moderate correlations (typically |r|≲0.3). Even for the strongest predictors there remained substantial spread in $\left|\Delta D_{b,v}^{\left(j\right)}\right|$ at fixed predictor values, indicating that these features explain only part of the voxel‐wise uncertainty and that the variability of the deltas tends to increase at higher nominal dose and dose‐gradient.

As a methods check, signed and absolute voxel‐level deltas defined using the Monte Carlo mode, median, or mean were all extremely highly correlated (Pearson r≥0.94; Table C7), indicating that all three capture the same underlying nominal‐versus‐perturbed discrepancy.

### 3.5 Threshold‐based QA of biopsy DVH criteria under localization uncertainty

#### 3.5.1 Monte Carlo pass probabilities and confidence classes

**FIGURE 5:**
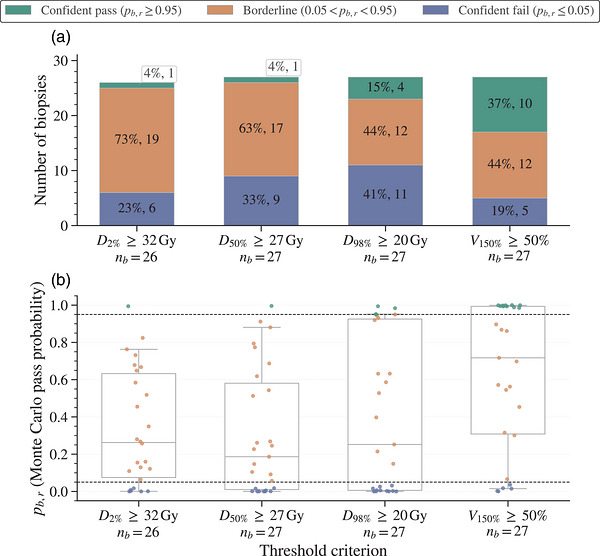
Cohort summary of Monte Carlo threshold classification for the four DVH‐based biopsy rules. (A) Fraction of biopsies classified as confident pass ($p_{b,r}\geq 0.95$), borderline ($0.05<p_{b,r}<0.95$), or confident fail ($p_{b,r}\leq 0.05$) for each rule under the fixed localization model. (B) Distributions of per‐biopsy pass probability $p_{b,r}$ with reference lines at 0.05 and 0.95 marking the confident fail and confident pass cutoffs.

Cohort‐level Monte Carlo summaries for the four DVH‐based biopsy rules in Equation (1) are shown in Figure 5 and Table C11. For each biopsy b and rule r, the pass probability $p_{b,r}$ was computed from the perturbed trials as in Equation (B26), and biopsy‐rule pairs were classified using the confidence classes defined in Section 2.7.

For the coverage metric $D_{98\%}\geq 20\;\mathrm{Gy}$, the median pass probability across biopsies was 0.25 (IQR 0.01‐0.93), with 4 of 27 biopsies (15%) classified as confident pass, 11 (41%) as confident fail, and 12 (44%) as borderline. For $D_{50\%}\geq 27\;\mathrm{Gy}$, pass probabilities were shifted toward lower values, with median 0.19 (IQR 0.01‐0.58); only 1 biopsy (4%) was a confident pass, 9 (33%) were confident fails, and 17 (63%) were borderline. For the hotspot dose metric $D_{2\%}\geq 32\;\mathrm{Gy}$, the median pass probability was 0.26 (IQR 0.08‐0.63). Among the 26 biopsies analyzed for this rule (one influential outlier excluded as prespecified in Section 2.7), 1 (4%) was a confident pass, 6 (23%) were confident fails, and 19 (73%) were borderline.

The hotspot volume rule $V_{150\%}\geq 50\%$ exhibited the highest fraction of confident passes: median $p_{b,r}=0.72$ (IQR 0.31‐0.99), with 10 of 27 biopsies (37%) classified as confident pass, 5 (19%) as confident fail, and 12 (44%) as borderline. Across rules, median nominal margins from Equation (B27) were modestly negative for the three dose criteria (approximately −1.2, −2.4, and −4.6Gy for $D_{98\%}$, $D_{50\%}$, and $D_{2\%}$, respectively) and strongly positive for $V_{150\%}$ (median 28.6 percentage points; Table C11). Thus, the coverage rules are rarely satisfied with high confidence and most biopsies remain borderline, whereas the $V_{150\%}\geq 50\%$ rule is comparatively robust but still leaves a substantial fraction of uncertain cases. These patterns reflect both the chosen DVH thresholds and the assumed localization model, rather than demonstrating intrinsically inadequate coverage in this cohort. In all rules, nominal passes either remained confident passes or shifted to the borderline class under Monte Carlo propagation; nominal fails did not flip directly to confident passes.

#### 3.5.2 Margin‐only logistic models

**FIGURE 6:**
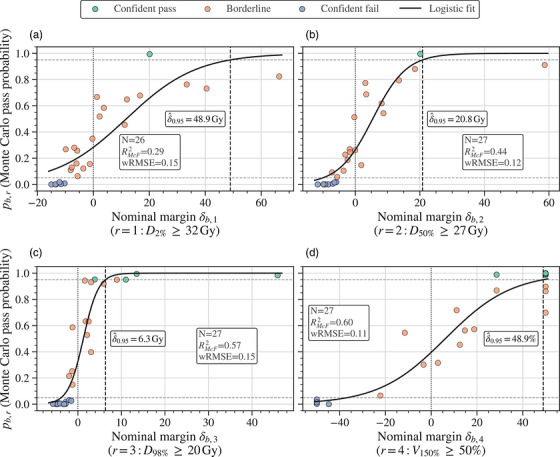
Pass probability $p_{b,r}$ versus nominal margin $\delta_{b,r}$ for each threshold rule r: (A) $D_{2\%}\geq 32\;\mathrm{Gy}$, (B) $D_{50\%}\geq 27\;\mathrm{Gy}$, (C) $D_{98\%}\geq 20\;\mathrm{Gy}$, and (D) $V_{150\%}\geq 50\%$. Points are per‐biopsy Monte Carlo estimates and curves are cohort‐level logistic fits using nominal margin as a single predictor. Horizontal reference lines indicate the 0.05 and 0.95 confidence cutoffs, and the vertical reference line marks the estimated nominal margin above threshold at which $p_{b,r}=0.95$ under the fitted model. For the $D_{2\%}$ rule, one influential biopsy was excluded (so N=26) as described in Section 2.7.

Logistic models relating Monte Carlo pass probabilities to nominal distance‐from‐threshold were fit using the margin‐only specification in Equation (2), with summaries in Figure 6 and Table C12. The fitted coefficients corresponded to odds ratios per Gy (or percentage point) in the expected direction: increasing $\delta_{b,r}$ increased the probability of passing. For example, for $D_{50\%}\geq 27\;\mathrm{Gy}$ the odds of passing increased by a factor of 1.21 per additional Gy of nominal margin (equivalently 2.60 per 5Gy), and for $D_{98\%}\geq 20\;\mathrm{Gy}$ the odds ratio per Gy was 1.78. McFadden $R^{2}$ values ranged from 0.29 for $D_{2\%}$ to 0.60 for $V_{150\%}$, indicating that nominal margin alone explained a substantial fraction of variability in $p_{b,r}$.

Under the fitted models, the nominal margins required to achieve $p_{b,r}=0.95$ were approximately 6.3Gy, 20.8Gy, and 48.9Gy for $D_{98\%}$, $D_{50\%}$, and $D_{2\%}$, and 48.9 percentage points for $V_{150\%}$ (Table C12). The corresponding 50%‐pass margins were much smaller (about 1‐12Gy or 6.2%). Margins of this magnitude are large compared with the 13.5Gy per‐fraction prescription and highlight how strongly localization uncertainty degrades biopsy‐scale DVH robustness.

For the $D_{2\%}$ rule, one biopsy exhibited extreme leverage on the logistic fit by Cook's distance. As prespecified in Section 2.7, this biopsy was excluded from both $D_{2\%}$‐specific logistic modelling and threshold QA summaries, so Tables C11 and C12 report N=26 for this rule.

#### 3.5.3 Secondary predictors and model comparison

To evaluate whether additional biopsy‐level characteristics improved prediction of $p_{b,r}$ beyond nominal margin, we extended the margin‐only model with a single biopsy‐specific predictor $g_{b,r}$ as in Equation (B31). Candidate predictors included nominal dose‐gradient magnitude, geometric and distance‐based features, and radiomics‐style descriptors summarized per biopsy (Appendix A). For each rule r and each candidate $g_{b,r}$, we fit the margin‐only model Equation (2) and the corresponding two‐predictor model on the same complete‐case subset and compared them using the change in AIC and a nested likelihood‐ratio test.

**FIGURE C1:**
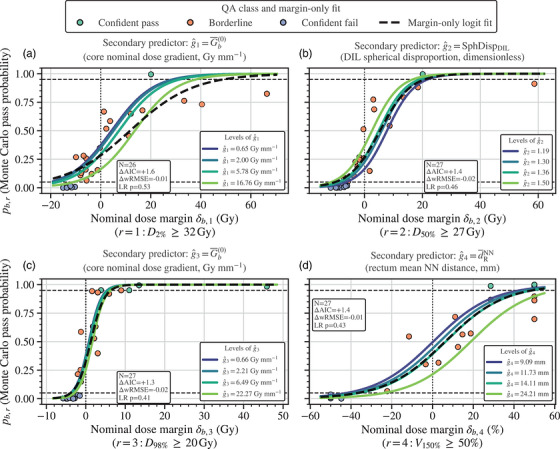
Secondary logistic modeling including nominal margin and the best secondary predictor for each threshold rule: (A) $D_{2\%}\geq 32\;\mathrm{Gy}$ with ${\hat{g}}_{1}={\bar{G}}_{b}^{\left(0\right)}$, (B) $D_{50\%}\geq 27\;\mathrm{Gy}$ with ${\hat{g}}_{2}={\mathrm{SphDisp}}_{\mathrm{DIL}}$, (C) $D_{98\%}\geq 20\;\mathrm{Gy}$ with ${\hat{g}}_{3}={\bar{G}}_{b}^{\left(0\right)}$, and (D) $V_{150\%}\geq 50\%$ with ${\hat{g}}_{4}={\bar{d}}_{R}^{\mathrm{NN}}$. Colored curves show the fitted two‐predictor logistic families at representative predictor levels, and the dashed black curve is the corresponding margin‐only model. This analysis is reported as a secondary model because adding the secondary predictor term did not appreciably improve fit by AIC or likelihood‐ratio tests (Table C13).

Across all four rules, adding the best secondary predictor produced only modest changes in fit. The best ΔAIC values were small and positive (from +1.3 to +1.6) and likelihood‐ratio p‐values ranged from 0.409 to 0.533, indicating no statistically compelling improvement over the margin‐only models (Table C13). The corresponding fitted two‐predictor response families are shown in Figure C1. The largest standardized change in the required 95%‐pass margin occurred for the hotspot‐volume rule $V_{150\%}\geq 50\%$, where increasing the rectum surface mean nearest‐neighbor distance from its 10th to 90th percentile increased the required margin by 18.5 percentage points, about 1.5 times the cohort‐median Monte Carlo SD of $V_{150\%}$. For the other rules, changes in ${\hat{\delta }}_{0.95}$ between low and high values of the best predictor were smaller than or comparable to the Monte Carlo noise scale $\sigma_{r}$. Thus, under the fixed localization uncertainty model, nominal distance‐from‐threshold $\delta_{b,r}$ appears to be the dominant determinant of threshold robustness for the biopsy DVH metrics considered, with additional dependence on geometry, DIL shape, or proximity to rectum remaining secondary.

**FIGURE 7:**
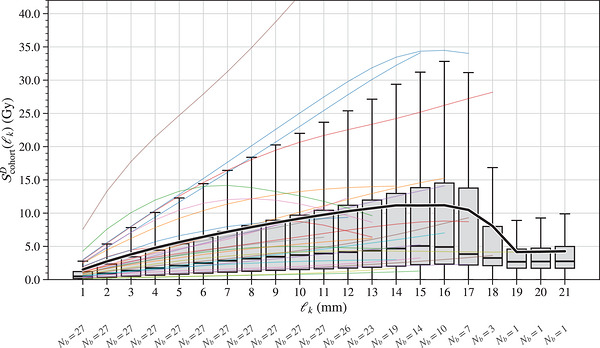
Absolute dose differences between all voxel pairs and Monte Carlo uncertainty trials at a given axial separation within each biopsy. Voxels were not compared between biopsies, only aggregated across them. The value $N_{p}$ below each length‐scale value indicates the total number of voxel‐pair samples at that separation, determined by the number of biopsies $N_{b}$ that are at least that long. The plateau in mean absolute difference at separations of about 1–1.5cm provides an approximate decorrelation scale for dose along the biopsy cores.

### 3.6 Intra‐core spatial heterogeneity and characteristic length scales

#### 3.6.1 Length‐scale dependence of voxel‐pair differences

**FIGURE C2:**
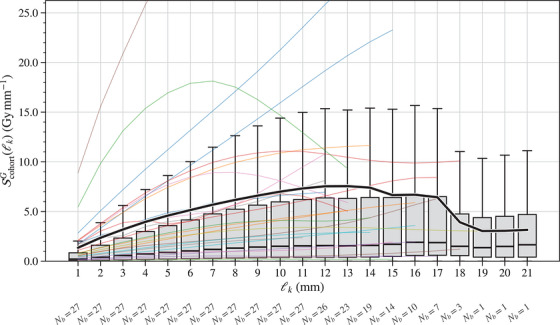
Absolute dose‐gradient magnitude differences between every voxel pair and Monte Carlo uncertainty trial at a given axial separation within each biopsy. Voxels were not compared between biopsies, only aggregated across them. The value $N_{p}$ below each length‐scale value indicates the total number of data points for that separation, determined by the number of biopsies $N_{b}$ that are at least that long. As for dose, mean absolute gradient differences increase with separation and plateau at separations of about 1‐1.5cm, consistent with a centimeter‐scale decorrelation length for dose gradient along the cores.

Intra‐core spatial heterogeneity was quantified by absolute voxel‐pair differences in dose and dose gradient as a function of axial separation $\ell_{k}$ (Figure 7 and C2). For each biopsy and each separation k, all voxel pairs separated by k voxels were considered across all Monte Carlo trials, and their absolute differences pooled across the cohort. The resulting distributions broadened rapidly with increasing separation for both dose and gradient. The mean absolute dose difference increased approximately linearly for small separations before gradually plateauing around separations of 1–1.5cm, where mean absolute differences were on the order of 10Gy. A similar pattern was observed for dose gradient, with mean absolute differences of several $\text{Gy}\;{\text{mm}}^{-1}$ at centimeter‐scale separations. Because the number of voxel pairs decreases with increasing separation, estimates at larger $\ell_{k}$ are based on fewer observations and are correspondingly more uncertain.

Taken together, these length‐scale analyses provide evidence that dose along a biopsy core decorrelates over distances on the order of 1–1.5cm under the combined influence of the HDR source arrangement and localization uncertainty. A typical 1–2cm biopsy track therefore spans multiple decorrelation lengths, so treating the entire core as having a single representative dose is not realistic. The apparent plateau in mean absolute differences at larger separations should be interpreted as an approximate decorrelation scale rather than a sharply defined cutoff.

#### 3.6.2 Voxel‐index heatmaps

**FIGURE C3:**
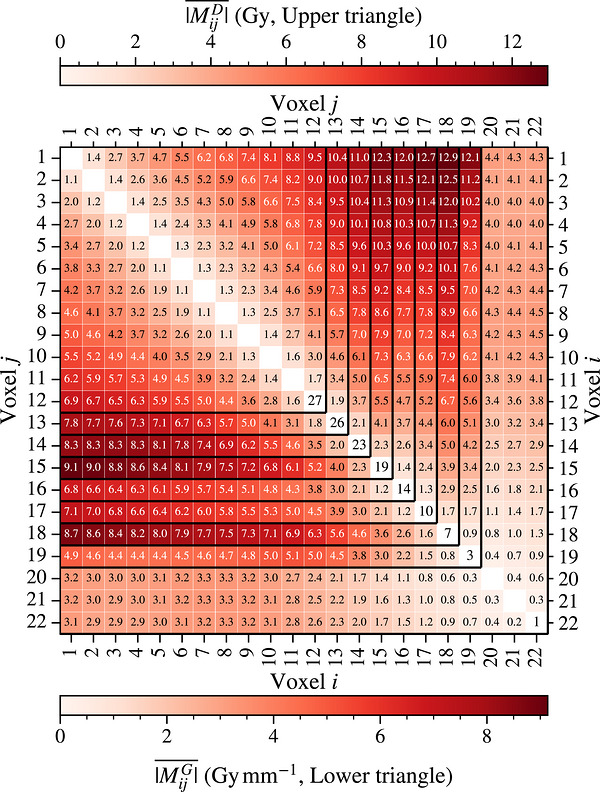
Heatmap displaying the mean absolute differences between voxel pairs (upper triangle: dose; lower triangle: dose‐gradient magnitude) of the pooled data across all 27 biopsies and Monte Carlo trials. The number of biopsies contributing to each mean in each block decreases as voxel‐pair separation increases because fewer cores are long enough to provide those pairs. For each biopsy and voxel‐index pair, the mean is taken over all localization trials.

Voxel‐index pair heatmaps provide a complementary view of intra‐core heterogeneity (Figure C3). In these plots, the upper triangle encodes the mean absolute dose difference between voxel indices i and j, and the lower triangle encodes the corresponding mean absolute difference in dose gradient, pooled across biopsies and trials. Near‐diagonal entries (small separations) show low mean absolute differences, whereas off‐diagonal entries (large separations) show substantially higher values. The number of biopsies contributing to each cell decreases with increasing separation, consistent with shorter cores providing fewer long‐range pairs.

Taken together, the length‐scale curves and voxel‐index heatmaps confirm that, while dose and gradient are locally smooth along each core, substantial variation can occur over centimeter‐scale distances. This further supports the need to treat biopsy dosimetry as spatially structured rather than assuming uniform exposure along the core, and it is consistent with the decorrelation length of approximately 1–1.5cm inferred from Figure 7.

### 3.7 Results summary

Across the cohort, these results show that (i) nominal voxel and biopsy doses differ from localization‐propagated quantities by several Gy on average, with nominal assignments mildly biased high; (ii) for the DVH criteria studied, the nominal distance from threshold largely determines robustness, and achieving a 95% chance of satisfying a rule under the fixed localization model can require nominal buffers of approximately 6‐21Gy for coverage metrics and nearly 50Gy or percentage points for hotspot metrics; and (iii) dose along a biopsy decorrelates over characteristic length scales of roughly 1–1.5cm. Together, these findings support treating biopsy DVH metrics as probabilistic quantities and reporting both nominal values and their Monte Carlo‐derived robustness when interpreting biopsy dosimetry in HDR prostate brachytherapy.

## 4 DISCUSSION

### 4.1 Overview

This work extends the previous voxel‐level localization‐uncertainty model for MR‐US guided prostate biopsies in HDR brachytherapy from dosimetric exemplar cores and probabilistic tissue classification to a cohort‐level, DVH‐style quality assurance framework.^31^, ^32^ Under a fixed but explicitly specified uncertainty model (isotropic Gaussian translations with σ=1.25mm plus an axial tissue‐deficit offset), we (i) characterized pooled voxel‐ and biopsy‐level dose and dose‐gradient distributions and the discrepancies between nominal and Monte Carlo‐propagated quantities, and (ii) translated biopsy DVH‐based robustness rules into pass probabilities as functions of nominal distance‐from‐threshold. In parallel, voxel‐pair analyses provided a cohort‐level estimate of the decorrelation length for dose along biopsies. Together, these results turn standard biopsy DVH criteria into probabilistic robustness rules under an explicit localization‐uncertainty model and quantify how nominal margin translates into robustness under that model.

### 4.2 Voxel‐ and biopsy‐level dose distributions

The pooled voxel‐wise dose and dose‐gradient distributions were broad, right‐skewed, and well described by log‐normal models (Figure 2 and Table C1). Biopsy voxels therefore sample a mixture of low‐gradient plateau regions and steep gradients near dwell positions, with local doses spanning a large dynamic range even within single cores. At the biopsy level, nominal core‐mean doses and gradients exceeded Monte Carlo medians on average, and biopsy‐specific 90% interval widths were wide for some cores (Tables C2 and C3), confirming substantial intra‐ and inter‐core heterogeneity.

Operationally, the localization‐uncertainty model rigidly perturbs the entire voxelized core, so the Monte Carlo ensemble samples a small tube of tissue around the recorded trajectory, with a transverse scale set by σ=1.25mm. The resulting biopsy‐level dose distributions are therefore better interpreted as summaries of the local dose microenvironment surrounding the sampled tissue, rather than as point estimates along an idealized line. This perspective is important for biological correlation studies, where paracrine, stromal, or mixed benign/malignant effects at millimeter scales may be more relevant than a single nominal biopsy dose.

### 4.3 Nominal versus uncertainty‐propagated assignments

Nominal voxel assignments differed meaningfully from localization‐perturbed realizations. Pooled over voxels and trials, the mean absolute per‐trial difference was about 7.6Gy for dose and $7.7\;\text{Gy}\;{\text{mm}}^{-1}$ for dose gradient, with 95th percentiles of ∼33Gy and $∼37\;\text{Gy}\;{\text{mm}}^{-1}$, respectively (Table C6). For uncertainty‐propagated summary deltas, typical absolute discrepancies remained nontrivial, on the order of a few Gy for dose and several $\text{Gy}\;{\text{mm}}^{-1}$ for gradient (Table C5). Common‐language effect sizes further showed a mild tendency for nominal values to exceed perturbed draws (probability ≈0.58 for dose and ≈0.55 for gradient), indicating a small upward bias in nominal voxel assignments.

At the same time, the mode, median, and mean Monte Carlo summaries were extremely highly correlated with each other (Pearson r≥0.94; Table C7), implying that they capture essentially the same underlying nominal‐versus‐perturbed discrepancy. The main shift in interpretation is therefore from purely nominal biopsy dosimetry to explicitly uncertainty‐propagated biopsy dosimetry; the choice among specific Monte Carlo summaries is secondary. For physics‐focused QA, nominal voxel and biopsy metrics remain natural descriptions of the planned configuration, but they should be accompanied by uncertainty‐propagated summaries such as biopsy‐level medians and IPR90 widths. For biology‐focused analyses that relate dose to histology, genomics, or Raman‐based endpoints, it will often be more informative to pair nominal metrics with a selected set of Monte Carlo‐propagated dose descriptors to form a vector of covariates.

### 4.4 Determinants of DVH‐threshold robustness

The DVH thresholds studied here ($D_{98\%}\geq 20\;\mathrm{Gy}$, $D_{50\%}\geq 27\;\mathrm{Gy}$, $D_{2\%}\geq 32\;\mathrm{Gy}$, and $V_{150\%}\geq 50\%$) should be viewed as illustrative biopsy‐scale rules spanning plausible coverage‐ and hotspot‐type behavior in this cohort, rather than as definitive clinical planning constraints for HDR prostate brachytherapy. Alternative DVH thresholds may be defined for other quality‐assurance protocols or research applications, including different HDR prescriptions and fractionation schemes. The observed mix of low and high Monte Carlo pass probabilities under these rules therefore indicates sensitivity of biopsy DVH classification under the localization uncertainty model and radiotherapy modality, rather than demonstrating inadequate planning. Many biopsy‐rule pairs fell into the borderline band, particularly for $D_{2\%}$ and $D_{50\%}$ (Figure 5 and Table C11), indicating cases in which threshold satisfaction is uncertainty‐sensitive and should be interpreted cautiously. This highlights the importance of treating biopsy dosimetry as a quantity that carries uncertainty and is therefore better described probabilistically under an explicit uncertainty model, rather than assuming that nominal values are robust representatives of the plausible biopsy dose distribution.

Despite this, nominal distance‐from‐threshold, $\delta_{b,r}$, captured most of the variability in pass probability. Margin‐only models achieved McFadden $R^{2}\approx 0.29$–0.60 across robustness rules (Figure 6 and Table C12) and yielded rule‐specific nominal buffers required to attain a 95% pass probability under the fixed uncertainty model: approximately 6Gy for $D_{98\%}\geq 20\;\mathrm{Gy}$, 21Gy for $D_{50\%}\geq 27\;\mathrm{Gy}$, 49Gy for $D_{2\%}\geq 32\;\mathrm{Gy}$, and 49% for $V_{150\%}\geq 50\%$. These values are best interpreted as biopsy‐scale robustness buffers for QA and interpretation, not as clinically prescriptive dose additions. Their large magnitude reflects the combination of millimeter‐scale localization perturbations and a spatially heterogeneous HDR dose field, under which single nominal biopsy assignments are not robust.

Adding biopsy‐level geometric, spatial, or radiomics‐style predictors did not materially improve prediction once nominal margin was included. Across rules, the best secondary predictors produced positive ΔAIC values and non‐significant likelihood‐ratio tests, and shifts in the required 95%‐pass margin were at most comparable to the inherent Monte Carlo variability $\sigma_{r}$ of the DVH metric $M_{b,r}$ (Table C13). In practical terms, this suggests that, under the present uncertainty model, nominal distance‐from‐threshold is the dominant determinant of robustness for the DVH metrics considered, and that more elaborate multiparameter QA rules provide limited additional benefit for this cohort.

In practical use, this framework is therefore best applied by reporting a nominal biopsy metric together with its Monte Carlo pass probability, and by treating borderline cases cautiously, rather than by attempting to enforce 95% robustness for every biopsy rule irrespective of nearby rectal or urethral constraints. For protocol design and retrospective QA, the fitted nominal margin‐to‐robustness relationships may support biopsy‐dose interpretation or inform biopsy‐selection strategies within already clinically acceptable dose distributions. Any prospective use to inform focal escalation would still require co‐optimization against standard target and OAR objectives, so the present margins should be interpreted primarily as biopsy‐scale uncertainty guidance rather than as standalone planning prescriptions.

### 4.5 Spatial heterogeneity and decorrelation length scales

Voxel‐pair analyses showed that absolute dose differences along a biopsy increase with axial separation and approach a plateau around 1–1.5cm, with mean absolute dose differences of order 10Gy at those scales (Figure 7). Dose‐gradient differences showed a similar separation dependence, reaching several $\text{Gy}\;{\text{mm}}^{-1}$ at centimeter‐scale separations. The reported 1–1.5cm scale describes how quickly biopsy voxels separated along the core cease to vary together under the combined influence of the superposed HDR field, biopsy orientation, and localization uncertainty. Local HDR dose‐gradient steepness contributes to this behavior, but does not determine it on its own; the observed length scale is better interpreted as an empirical along‐core spatial dependence scale under the stated uncertainty model. Part of this behavior may also reflect biopsy orientation: because the cores in this cohort were predominantly aligned along the superior‐inferior axis, with only modest tilt in some cases, and were therefore oriented broadly in the same direction as the implanted HDR catheters, along‐core separations often sampled a component of the HDR dose field that can vary more gradually than the steepest local gradients transverse to individual dwell positions. This is also consistent with Figure 7, where the biopsy‐specific curves show substantial heterogeneity, with some cores exhibiting steeper dose‐difference growth with separation than others.

At short separations, near‐diagonal voxel pairs remain substantially more similar than distant pairs, as seen in both the length‐scale curves and the voxel‐index heatmaps (Figures 7 and C3). Because only the longest biopsies contribute at larger separations, the number of contributing voxel pairs decreases rapidly beyond about 1cm, so estimates near and beyond the apparent plateau should be interpreted as approximate and sample‐size limited. These findings are consistent with earlier exemplar‐core analyses in the same cohort,^31^ and together support a decorrelation length of approximately 1–1.5cm for dose along HDR biopsy tracks. A typical 1–2cm biopsy therefore spans multiple decorrelation lengths, so a single representative dose is unlikely to adequately describe the full core. For analyses that attempt to relate spatially resolved pathology or imaging to dose, this argues for local, spatially explicit dose summaries (for example, by voxel or short segments) rather than assuming uniform exposure along the core.

### 4.6 Limitations

Several limitations should be acknowledged. First, the localization‐uncertainty model is a first‐order approximation: a rigid 3D translation with an isotropic Gaussian prior plus an axial tissue‐deficit offset. This preserves biopsy connectivity and is aligned with previous work on this cohort,^31^ but does not capture anisotropic errors, needle bending, patient‐specific deformation, or fraction‐to‐fraction anatomical changes. More complex, deformable, or patient‐specific models could alter both the magnitude and directionality of propagated dose changes and would require independent validation and additional model development.

A related limitation is that MRI‐derived lesion definition enters the present analysis only upstream, through DIL delineation, MRI‐TRUS target transfer, and any resulting effects on cognitive targeting or boost‐oriented planning, rather than through the biopsy‐to‐dose sampling step itself. The clinical dose mapping analyzed here was performed entirely in the ultrasound/planning frame on the HDR dose lattice. The 1mm slice thickness of the T2‐weighted and diffusion‐weighted/ADC sequences, together with any associated partial‐volume effects, therefore primarily affects lesion delineation and target‐transfer accuracy, with indirect consequences for lesion localization and treatment planning, rather than the direct interpolation of dose to biopsy voxels.

Separately, deformation differences between endorectal‐coil MRI and TRUS were not represented here as an explicit deformable transform. This was not only a simplification of the uncertainty model, but also reflects the scope of the present analysis: for biopsy‐to‐dose assignment, the relevant quantities are the already calculated clinical dose field and the indicated biopsy position in the planning frame, neither of which is directly altered by MRI‐derived lesion‐definition uncertainty. Such deformation effects therefore enter this study only indirectly, through upstream lesion delineation, MRI‐TRUS target transfer, and any resulting effects on planning or cognitive targeting, rather than through the direct mapping of dose to reconstructed biopsy voxels. This approximation is therefore more consequential for lesion localization and treatment planning than for downstream assignment of the calculated dose field to the biopsy in the planning frame.

Another limitation is that the analysis is based on 27 biopsies from 15 patients at a single center, with a specific HDR prescription and workflow. While each biopsy is supported by a large number of Monte Carlo trials, the number of independent biopsy samples is modest, which limits power to detect subtler effects of biopsy geometry or plan‐specific dose‐field variation on robustness and cautions against direct extrapolation to other institutions or fractionation schemes without recalibration. Extending the framework to cumulative multi‐fraction dose or to biopsies obtained after the second fraction would require explicit registration of dose fields across plans, likely using deformable registration techniques.

The set of geometric, dosimetric, and radiomic predictors considered here, although broad, is not exhaustive. In larger cohorts, additional treatment‐level and plan‐specific variables, such as catheter number and arrangement, source activity, dwell‐time structure, or biopsy trajectory relative to the implanted catheter geometry, may capture further variation in threshold robustness. The secondary‐predictor analysis should therefore be viewed as exploratory.

### 4.7 Future work

Future extensions should focus first on refining the uncertainty model and validating it prospectively. In particular, patient‐specific or deformable registration‐error models could be compared against the present first‐order rigid‐plus‐axial formulation, and the resulting biopsy‐scale robustness metrics could be reassessed under alternative uncertainty assumptions. The observed decorrelation length also motivates spatial models, including Gaussian process regression, that treat dose along the biopsy track as a correlated process rather than as independent voxel values.

A second direction is integration with other biopsy‐localized measurements. The present DVH‐based robustness framework could be combined with probabilistic tissue‐classification outputs and extended to MR‐derived quantities such as ADC or T2, yielding a unified uncertainty framework for biopsy tissue class, dosimetry, and imaging biomarkers. In larger cohorts, the same descriptors could also support exploratory clustering to identify recurring dosimetric phenotypes and relate them to histopathology, Raman spectroscopy, or genomic endpoints.

## 5 CONCLUSION

In conclusion, this study shows that, under an explicit millimeter‐scale localization‐uncertainty model, (i) nominal voxel and biopsy doses differ from Monte Carlo‐propagated quantities by several Gy on average and are mildly biased high, (ii) for the biopsy‐scale DVH robustness rules studied here, nominal distance‐from‐threshold is the dominant determinant of robustness and substantial nominal buffers are required to attain high pass probabilities, and (iii) dose along biopsy cores decorrelates over distances of order 1–1.5cm. These findings support the use of Monte Carlo‐propagated, threshold‐based reporting of biopsy dosimetry in HDR prostate brachytherapy, particularly for probabilistic quality assurance and biopsy‐based dose‐biology studies. Together, these results provide a foundation for future work linking localized dose with probabilistic tissue classification and MR‐derived biomarkers in biopsy‐based correlation studies.

## AUTHOR CONTRIBUTIONS

M. Muscat conceived the study, developed the localization uncertainty and dosimetry framework, implemented all analysis code, performed the data processing and statistical analyses, and drafted the manuscript. N. Becker supervised the project, contributed to study conception and design, and provided ongoing feedback on the analysis and manuscript. A. Jirasek contributed to study design, methodological guidance, and critical revision of the manuscript. J. Crook oversaw clinical aspects of the study, including biopsy acquisition and interpretation of HDR brachytherapy practice, and contributed to clinical interpretation and critical revision of the manuscript. J. Andrews provided statistical and methodological input through project meetings. All authors reviewed and approved the final manuscript.

## CONFLICT OF INTEREST STATEMENT

The authors declare no conflicts of interest.

## ETHICAL APPROVAL

This study was conducted in accordance with institutional research ethics approvals under protocols H17‐02904, H24‐00948 and H23‐02872 and clinical trial NCT02692105. All patients provided written informed consent for participation in the clinical trial and for the use of their imaging and dosimetric data for research.

## Data Availability

The imaging data used in this study consist of clinical DICOM datasets from patients treated at BC Cancer that were de‐identified and processed using the authors' analysis pipeline. The original DICOM files and derived patient‐level datasets are not available in a public repository because they contain protected health information and are subject to institutional and provincial privacy regulations. De‐identified aggregate data and analysis outputs may be made available from the corresponding author on reasonable request and with prior approval from BC Cancer and the University of British Columbia research ethics boards.

## Footnotes

* For each set of trialwise values we fitted a univariate Gaussian kernel density estimate with bandwidth chosen by Scott's rule, $h=\hat{\sigma }\;n^{-1/5}$, where $\hat{\sigma }$ is the sample standard deviation and n the number of trials. The density was evaluated on a regular grid of 10000 points over the observed range and the mode was taken as the grid point with maximal estimated density. When the values were numerically constant we set the mode equal to that common value, and if the kernel‐density fit failed we defaulted to the sample median.

† Cook's distance was computed from a margin‐only logistic regression of biopsy‐level pass probability on the nominal distance‐from‐threshold, with binomial weights given by the number of Monte Carlo trials per biopsy. We used the common 4/n rule‐of‐thumb cutoff (with n biopsies for this rule); the biopsy with the largest Cook's distance exceeded this threshold and was therefore excluded from all $D_{2\%}$‐specific logistic and descriptive summaries.

## APPENDIX A BIOPSY‐LEVEL GEOMETRIC, SPATIAL, AND RADIOMICS‐STYLE PREDICTORS

This appendix summarizes the biopsy‐assigned geometric and radiomic predictors considered in the secondary (margin + predictor) logistic models (Section 2.7) as well as in the Δ assessments (Section 2.5). Predictors are defined either per biopsy (b) or per biopsy voxel (v), following the index conventions (explained below).

### A.1 General segmentation and feature‐calculation framework

All structure‐based predictors (for the biopsy, the targeted dominant intraprostatic lesion (DIL), and the prostate) were derived from applying slice‐wise point‐in‐polygon testing, binary masks on an isotropic 3D grid (1mm×1mm×1mm voxelization) obtained from clinical segmentations, or meshing tools on interpolated segmentations.^33^ For each mask, an in‐house meshing and voxelization pipeline was used to compute:

- the enclosed volume by counting voxels and multiplying by voxel volume, with mesh‐based checks for consistency; and
- the surface area from a triangulated surface mesh extracted from the interpolated segmentations.

Shape descriptors such as surface‐area‐to‐volume ratio, sphericity, compactness, spherical disproportion, maximum 3D diameter, and principal‐component (PCA) axis lengths and derived ratios were then computed from these basic volume and surface‐area quantities, or from slice‐wise point‐in‐polygon analyses, using an in‐house radiomics‐style pipeline informed by standard radiomics terminology.^35^, ^36^

### A.2 Biopsy geometry

For each biopsy core, **core length** $L_{b}$ (mm) was defined as the physical length of the reconstructed core, measured along the core axis as the distance between the most proximal and most distal sampled points in the planning frame. This quantity describes the overall axial extent of the sampled tissue and was retained as a simple geometric predictor in the reported formal scans.

### A.3 Distances to surrounding structures

Distances were computed between the biopsy and four surrounding structures: the targeted DIL, prostate, rectum, and urethra. Each structure was represented by a contained‐point binary mask; triangulated surface meshes were used for surface‐area estimation, and reconstructed boundary point clouds were used for nearest‐neighbor distance calculations.

For each biopsy‐organ pair:

- **Nearest‐neighbor surface distance (mm)**: the mean signed Euclidean distance, across sampled biopsy points and Monte Carlo trials, from the biopsy to the nearest point on the reconstructed organ boundary representation. Negative values indicate sampled points lying within the structure and positive values indicate sampled points lying outside it. These mean distances were used as nearest‐neighbor (NN) distance predictors for each organ.
- **Point‐to‐centroid distance (mm)**: the mean Euclidean distance, across sampled biopsy points and Monte Carlo trials, from the biopsy to the organ centroid.

In addition, a **normalized biopsy‐prostate centroid distance** was defined by dividing the corresponding biopsy‐to‐prostate centroid distance summary by a characteristic prostate size, taken as the mean of the prostate left‐right, anterior‐posterior, and superior‐inferior dimensions evaluated at the prostate centroid. In the voxel‐level bias analyses this produced a voxel‐wise normalized distance, whereas in the threshold‐QA analyses the corresponding biopsy‐level mean prostate‐centroid distance was normalized by the same quantity.

### A.4 DIL and prostate radiomics‐style shape features

For both the targeted DIL and the whole prostate, an in‐house set of radiomics‐style 3D shape descriptors was computed from the contained‐point binary masks and reconstructed surface meshes. The terminology below follows standard radiomics usage,^35^, ^36^ but the features were generated by the present custom pipeline rather than by direct PyRadiomics extraction:

- **Volume** (${\mathrm{mm}}^{3}$): total enclosed volume of the structure.
- **Surface area** (${\mathrm{mm}}^{2}$): area of the triangulated surface.
- **Surface‐area‐to‐volume ratio** (${\mathrm{mm}}^{-1}$): ratio of surface area to volume.
- **Sphericity**: dimensionless measure of how close the structure is to a perfect sphere, computed from volume and surface area.
- **Compactness and spherical disproportion**: dimensionless shape measures computed from volume and surface area using standard radiomics‐style formulas.
- **Maximum 3D diameter (mm)**: maximum Euclidean distance between any two points in the structure point cloud.
- **PCA‐based axes and derived ratios**: principal‐component analysis of the binary‐mask point cloud was used to obtain three orthogonal principal directions together with the corresponding point‐cloud spans along those directions. These PCA‐derived span and ratio summaries were retained as custom shape descriptors, along with in‐house equivalent‐ellipsoid and elongation/flatness summaries.

These shape descriptors, together with the biopsy geometry and distance‐based quantities above, formed the broader feature pool used in the voxel‐level delta‐bias analyses described in Section 2.5. A restricted biopsy‐level subset of these quantities was then used as candidate secondary predictors in the margin‐plus‐predictor logistic models described in Section 2.7.

#### Index conventions

Throughout, the subscript b indexes biopsies and v indexes biopsy voxels. Quantities written as $X_{b,v}$ are defined per voxel, whereas $X_{b}$ denotes a biopsy‐level summary (typically an average over voxels in core b). Structure‐level quantities (e.g. prostate or DIL features) carry only a structure subscript (P, DIL, R, U) and no b or v. An overline $\bar{\left(\cdot \right)}$ indicates an average over Monte Carlo trials, and where a different averaging operation is used (e.g. averaging over voxels along a core to form ${\bar{G}}_{b}^{\left(0\right)}$), this is stated explicitly in the corresponding definition.

### List of continuous predictors used in the formal scans

The items below summarize the substantive continuous predictor families used in the reported delta‐bias and threshold‐QA analyses. The voxel‐level delta‐bias scans used a broader scalar‐column sweep, whereas the threshold‐QA margin‐correlation and margin‐plus‐secondary‐logit analyses used a restricted biopsy‐level subset consisting of nominal core‐mean gradient, normalized biopsy‐prostate centroid distance, biopsy length, organ centroid and NN distance summaries, and DIL/prostate volume‐, surface‐, compactness‐, sphericity‐, maximum‐diameter‐, and PCA‐span descriptors. Internal identifiers, categorical location labels, and other immaterial raw component fields are not listed here. For each predictor we give its name, symbol (where applicable), units, and a brief description.

**Nominal voxel dose** $D_{b,v}^{\left(0\right)}$ (Gy): Nominal dose assigned to biopsy voxel (b,v) on the clinical HDR dose lattice.
**Nominal voxel dose gradient** $G_{b,v}^{\left(0\right)}$ (Gy ${\mathrm{mm}}^{-1}$): Normed nominal dose‐gradient magnitude at biopsy voxel (b,v).
**Nominal core‐mean gradient** ${\bar{G}}_{b}^{\left(0\right)}\;\left(\text{Gy}\;{\text{mm}}^{-1}\right)$: Nominal core‐averaged (across voxels) dose gradient along the biopsy trajectory for Monte Carlo trial 0.
**Normalized biopsy‐prostate centroid distance** ${\bar{d}}_{P,v}^{\mathrm{norm},\mathrm{cen}}$ (dimensionless): In voxel‐level bias analyses, the voxel‐wise mean biopsy‐point‐to‐prostate‐centroid distance normalized by the mean prostate LR/AP/SI dimensions at the prostate centroid. In the threshold‐QA models, the corresponding biopsy‐level predictor uses the biopsy‐level mean prostate‐centroid distance normalized by the same prostate size.
**DIL centroid distance (mean)** ${\bar{d}}_{\mathrm{DIL}}^{\mathrm{cen}}\;\left(mm\right)$: Mean biopsy‐point‐to‐centroid distance to the targeted DIL, averaged across Monte Carlo trials.
**Prostate centroid distance (mean)** ${\bar{d}}_{P}^{\mathrm{cen}}\;\left(mm\right)$: Mean biopsy‐point‐to‐centroid distance to the prostate, averaged across Monte Carlo trials.
**Rectum centroid distance (mean)** ${\bar{d}}_{R}^{\mathrm{cen}}\;\left(mm\right)$: Mean biopsy‐point‐to‐centroid distance to the rectum, averaged across Monte Carlo trials.
**Urethra centroid distance (mean)** ${\bar{d}}_{U}^{\mathrm{cen}}\;\left(mm\right)$: Mean biopsy‐point‐to‐centroid distance to the urethra, averaged across Monte Carlo trials.
**DIL NN distance (mean)** ${\bar{d}}_{\mathrm{DIL}}^{\mathrm{NN}}\;\left(mm\right)$: Mean signed nearest‐neighbor distance between sampled biopsy points and the targeted DIL boundary representation.
**Prostate NN distance (mean)** ${\bar{d}}_{P}^{\mathrm{NN}}\;\left(mm\right)$: Mean signed nearest‐neighbor distance between sampled biopsy points and the prostate boundary representation.
**Rectum NN distance (mean)** ${\bar{d}}_{R}^{\mathrm{NN}}\;\left(mm\right)$: Mean signed nearest‐neighbor distance between sampled biopsy points and the rectum boundary representation.
**Urethra NN distance (mean)** ${\bar{d}}_{U}^{\mathrm{NN}}\;\left(mm\right)$: Mean signed nearest‐neighbor distance between sampled biopsy points and the urethra boundary representation.
**Biopsy length** $L_{b}\;\left(mm\right)$: Physical length of the biopsy core.
**DIL volume** $V_{\mathrm{DIL}}\;\left(mm^{3}\right)$: Volume of the targeted DIL.
**DIL surface area** $A_{\mathrm{DIL}}\;\left(mm^{2}\right)$: Surface area of the targeted DIL.
**DIL surface‐area‐to‐volume ratio** $A_{\mathrm{DIL}}/V_{\mathrm{DIL}}\;\left(mm^{-1}\right)$: Surface‐area‐to‐volume ratio of the targeted DIL.
**DIL sphericity** ${\mathrm{Sphericity}}_{\mathrm{DIL}}$ (dimensionless): Sphericity of the targeted DIL.
**DIL compactness 1** $\mathrm{Compactness}1_{\mathrm{DIL}}$ (dimensionless).
**DIL compactness 2** $\mathrm{Compactness}2_{\mathrm{DIL}}$ (dimensionless).
**DIL spherical disproportion** ${\mathrm{SphDisp}}_{\mathrm{DIL}}$ (dimensionless).
**DIL maximum 3D diameter** $D_{\mathrm{DIL}}^{\max}\;\left(mm\right)$: Maximum 3D diameter of the targeted DIL.
**DIL PCA major axis** $\lambda_{\mathrm{DIL},\mathrm{major}}^{\mathrm{PCA}}\;\left(mm\right)$: Largest PCA‐derived point‐cloud span of the DIL.
**DIL PCA minor axis** $\lambda_{\mathrm{DIL},\mathrm{minor}}^{\mathrm{PCA}}\;\left(mm\right)$: Intermediate PCA‐derived point‐cloud span of the DIL.
**DIL PCA least axis** $\lambda_{\mathrm{DIL},\mathrm{least}}^{\mathrm{PCA}}\;\left(mm\right)$: Smallest PCA‐derived point‐cloud span of the DIL.
**DIL equivalent‐ellipsoid axes** $a_{\mathrm{DIL},\mathrm{maj}}$, $a_{\mathrm{DIL},\min}$, $a_{\mathrm{DIL},\mathrm{least}}\;\left(mm\right)$: PCA‐derived equivalent‐ellipsoid axis lengths reported by the in‐house DIL shape‐feature pipeline.
**DIL elongation and flatness** ${\mathrm{Elongation}}_{\mathrm{DIL}}$, ${\mathrm{Flatness}}_{\mathrm{DIL}}$ (dimensionless): In‐house PCA‐derived elongation and flatness summaries of the DIL.
**DIL dimensions at centroid** $L_{\mathrm{DIL}}^{\mathrm{LR}}$, $L_{\mathrm{DIL}}^{\mathrm{AP}}$, $L_{\mathrm{DIL}}^{\mathrm{SI}}\;\left(mm\right)$: DIL extent in left‐right, anterior‐posterior, and superior‐inferior directions at the DIL centroid.
**Normalized DIL‐prostate centroid distance** $d_{\mathrm{DIL},\mathrm{prostate}}^{\mathrm{norm}}$ (dimensionless): Distance between the DIL and prostate centroids, normalized by the characteristic prostate size (mean LR/AP/SI dimensions).
**DIL superior‐inferior arclength** $L_{\mathrm{DIL}}^{\mathrm{SI},\mathrm{arc}}\;\left(mm\right)$: Superior‐inferior arclength of the DIL.
**Prostate volume** $V_{P}\;\left(mm^{3}\right)$: Prostate volume.
**Prostate surface area** $A_{P}\;\left(mm^{2}\right)$: Prostate surface area.
**Prostate surface‐area‐to‐volume ratio** $A_{P}/V_{P}\;\left(mm^{-1}\right)$: Surface‐area‐to‐volume ratio of the prostate.
**Prostate sphericity** ${\mathrm{Sphericity}}_{P}$ (dimensionless).
**Prostate compactness 1** $\mathrm{Compactness}1_{P}$ (dimensionless).
**Prostate compactness 2** $\mathrm{Compactness}2_{P}$ (dimensionless).
**Prostate spherical disproportion** ${\mathrm{SphDisp}}_{P}$ (dimensionless).
**Prostate maximum 3D diameter** $D_{P}^{\max}\;\left(mm\right)$.
**Prostate PCA major axis** $\lambda_{P,\mathrm{major}}^{\mathrm{PCA}}\;\left(mm\right)$: Largest PCA‐derived point‐cloud span of the prostate.
**Prostate PCA minor axis** $\lambda_{P,\mathrm{minor}}^{\mathrm{PCA}}\;\left(mm\right)$: Intermediate PCA‐derived point‐cloud span of the prostate.
**Prostate PCA least axis** $\lambda_{P,\mathrm{least}}^{\mathrm{PCA}}\;\left(mm\right)$: Smallest PCA‐derived point‐cloud span of the prostate.
**Prostate equivalent‐ellipsoid axes** $a_{P,\mathrm{maj}}$, $a_{P,\min}$, $a_{P,\mathrm{least}}\;\left(mm\right)$: PCA‐derived equivalent‐ellipsoid axis lengths reported by the in‐house prostate shape‐feature pipeline.
**Prostate elongation and flatness** ${\mathrm{Elongation}}_{P}$, ${\mathrm{Flatness}}_{P}$ (dimensionless): In‐house PCA‐derived elongation and flatness summaries of the prostate.
**Prostate dimensions at centroid** $d_{\mathrm{LR},\mathrm{prostate}},d_{\mathrm{AP},\mathrm{prostate}},d_{\mathrm{SI},\mathrm{prostate}}\;\left(mm\right)$: Prostate extent in left‐right, anterior‐posterior, and superior‐inferior directions at the prostate centroid.
**Prostate superior‐inferior arclength** $L_{P}^{\mathrm{SI},\mathrm{arc}}\;\left(mm\right)$: Superior‐inferior arclength of the prostate.
**Prostate mean dimension at centroid** ${\bar{d}}_{\mathrm{prostate}}\;\left(mm\right)$: Mean of the three orthogonal dimensions at the prostate centroid.

## APPENDIX B MATHEMATICAL FORMULATION OF VOXEL‐LEVEL DOSIMETRY AND QA METRICS

This appendix gathers the mathematical details of the voxel‐, biopsy‐, and cohort‐level framework, including interpolation, the localization‐uncertainty model, delta metrics, DVH definitions, QA thresholds, logistic models, and voxel‐pair analyses. The notation is consistent with the main text, where the same quantities are introduced in a descriptive manner.

### B.1 Dose‐gradient magnitude and interpolation

Clinical HDR dose was available as a rectilinear lattice $D(\vec{x})$ on a regular grid. The dose‐gradient magnitude was defined as

(B1)
$$G\left(\vec{x}\right)\equiv ∥\nabla D\left(\vec{x}\right)∥,$$

and computed using finite differences on the background dose lattice.

For any query point $\vec{x}$, dose was sampled using four‐nearest‐ neighbor inverse‐distance weighting (IDW) with power p=1:

(B2)
$$\hat{D}\left(\vec{x}\right)=\frac{\sum_{i=1}^{4}D_{\mathrm{NN},i}/d_{i}}{\sum_{i=1}^{4}1/d_{i}},$$

where, $D_{\mathrm{NN},i}$ are the four nearest lattice values, $d_{i}\equiv ∥\vec{x}-{\vec{x}}_{\mathrm{NN},i}∥$ are the corresponding Euclidean distances, and ${\vec{x}}_{\mathrm{NN},i}$ are the coordinates of the four nearest lattice points. If any $d_{i}=0$, we set $\hat{D}\left(\vec{x}\right)=D_{\mathrm{NN},i}$ for that coincident lattice point.

The same IDW rule was applied to sample $G(\vec{x})$, denoted $\hat{G}\left(\vec{x}\right)$, with the same coincident‐point convention.

### B.2 Localization‐uncertainty model

Monte Carlo trials were indexed by

(B3)
$$t\in \left\{0,1,\cdots ,N_{T}\right\},\;N_{T}=10,000,$$

where, t=0 is the nominal (unshifted) case and $t=1,\cdots ,N_{T}$ are localization‐perturbation trials.

#### Rigid translation

For each localization‐perturbation trial t≥1, a single translation vector was sampled from an isotropic normal distribution:

(B4)
$${\vec{T}}_{t}∼N\left(\vec{0},\sigma^{2}I\right),\;\sigma =1.25\;\mathrm{mm}.$$

The same ${\vec{T}}_{t}$ was applied to all voxels of a biopsy in trial t, preserving tissue connectivity under a rigid‐motion prior.

#### Axial tissue‐deficit offset

To account for mismatch between the needle compartment length $l_{c}$ and the extracted tissue length $l_{a}$, an independent offset along the core axis $\hat{c}$ was sampled for each trial:

(B5)
$$\begin{array}{cc} X_{l,t} & ∼U\;\left(0,\max(l_{c}-l_{a},0)\right),\\ {\vec{T}}_{t} & ={\vec{T}}_{t}+X_{l,t}\;\hat{c},\;{\vec{T}}_{0}=\vec{0}. \end{array}$$

#### Translated coordinates and sampled dose

For biopsy b, voxel v, and trial t, the translated voxel centre is

(B6)
$${\vec{x}}_{b,v}\left(t\right)={\vec{x}}_{b,v}\left(0\right)+{\vec{T}}_{t},$$

and the sampled dose and gradient magnitude are

(B7)
$$D_{b,v}^{\left(t\right)}\equiv \hat{D}\left({\vec{x}}_{b,v}\left(t\right)\right),\;G_{b,v}^{\left(t\right)}\equiv \hat{G}\left({\vec{x}}_{b,v}\left(t\right)\right).$$

For each voxel we obtain Monte Carlo sets ${\left\{D_{b,v}^{\left(t\right)}\right\}}_{t=0}^{N_{T}}$ and ${\left\{G_{b,v}^{\left(t\right)}\right\}}_{t=0}^{N_{T}}$.

### B.3 Multi‐level pooling and biopsy‐level summaries

#### Voxel level

For biopsy b and voxel v, define the trialwise perturbed samples

(B8)
$$D_{b,v}\equiv {\left\{D_{b,v}^{\left(t\right)}\right\}}_{t=1}^{N_{T}},\;G_{b,v}\equiv {\left\{G_{b,v}^{\left(t\right)}\right\}}_{t=1}^{N_{T}}.$$

Monte Carlo propagated voxel‐level summaries (mean, median, quantiles, standard deviation, and KDE‐based mode) were computed from $D_{b,v}$ and $G_{b,v}$.

#### Biopsy level

Within biopsy b, voxelwise values were pooled across voxels to form biopsy‐level pooled samples:

(B9)
$$D_{b}\equiv \overset{n_{b}}{\underset{v=1}{⊎}}D_{b,v},\;G_{b}\equiv \overset{n_{b}}{\underset{v=1}{⊎}}G_{b,v},$$

where, ⊎ denotes pooling by concatenation (multiset union), so repeated values are retained.

Nominal biopsy‐level core‐averaged dose and dose gradient were defined as

(B10)
$${\bar{D}}_{b}^{\left(0\right)}\equiv \frac{1}{n_{b}}\sum_{v=1}^{n_{b}}D_{b,v}^{\left(0\right)},\;{\bar{G}}_{b}^{\left(0\right)}\equiv \frac{1}{n_{b}}\sum_{v=1}^{n_{b}}G_{b,v}^{\left(0\right)}.$$

Throughout, we refer to ${\bar{D}}_{b}^{\left(0\right)}$ and ${\bar{G}}_{b}^{\left(0\right)}$ as the nominal core‐averaged dose and dose gradient, respectively.

#### Cohort level

Let B denote the number of biopsies. Pooled cohort distributions were formed by pooling biopsy‐level collections:

(B11)
$$\begin{array}{cc} D_{\mathrm{cohort}} & \equiv \overset{B}{\underset{b=1}{⊎}}D_{b},\\ G_{\mathrm{cohort}} & \equiv \overset{B}{\underset{b=1}{⊎}}G_{b}. \end{array}$$

Cohort‐level means and standard deviations reported in the Results and Tables are then computed across the corresponding per‐biopsy quantities (for example, across the B=27 values ${\left\{{\bar{D}}_{b}^{\left(0\right)}\right\}}_{b=1}^{B}$).

### B.4 Parametric histogram fits and log‐normal family

For the cohort‐pooled voxel‐level histograms we considered a set of standard continuous distributions: truncated normal, log‐normal, gamma, Weibull, exponential, Pareto, Rice, generalized gamma, generalized normal, and skew‐normal. For each metric and candidate family, parameters were estimated by maximum likelihood and models were compared using Akaike's information criterion (AIC). When the log‐normal family provided the best fit, we used a three‐parameter form with shape κ>0, location ξ∈R, and scale β>0.

Let X denote either dose or dose‐gradient magnitude. We write,

(B12)
X∼LogNormal(κ,ξ,β)

if,

(B13)
$$Y=\ln\;\left(\frac{X-\xi }{\beta }\right)∼N\left(0,\kappa^{2}\right),$$

so that the probability density of X is,

(B14)
$$\begin{array}{cc} f_{X}\left(x|\kappa ,\xi ,\beta \right) & =\frac{1}{\left(x-\xi \right)\;\kappa \sqrt{2\pi }}\\ & \;\times \exp\;\left\{-\frac{1}{2\kappa^{2}}{\left[\ln\;\left(\frac{x-\xi }{\beta }\right)\right]}^{2}\right\}, \end{array}\;x>\xi .$$

Under this parameterization, the median and mean of X are:

(B15)
$$\begin{array}{cc} \mathrm{median}(X) & =\xi +\beta , \end{array}$$

(B16)
$$\begin{array}{cc} \mathrm{mean}(X) & =\xi +\beta \;\exp\;\left(\frac{1}{2}\kappa^{2}\right). \end{array}$$

In the cohort histograms, the fitted log‐normal curves are therefore reported in terms of the triplet (κ,ξ,β), corresponding to shape, location, and scale, respectively.

### B.5 Delta metrics for nominal versus Monte Carlo‐propagated dose

#### Voxelwise statistical‐summary deltas

Let $D_{b,v}^{\left(j\right)}$ be a voxel summary computed from ${\left\{D_{b,v}^{\left(t\right)}\right\}}_{t=1}^{N_{T}}$, with j∈{mode,Q50,mean}. Summary deltas are

(B17)
$$\Delta D_{b,v}^{\left(j\right)}\equiv D_{b,v}^{\left(0\right)}-D_{b,v}^{\left(j\right)},\;\Delta G_{b,v}^{\left(j\right)}\equiv G_{b,v}^{\left(0\right)}-G_{b,v}^{\left(j\right)}.$$

For each summary type j, cohort‐level pooled collections were formed as

(B18)
$$\begin{array}{cc} D_{\Delta ,\mathrm{cohort}}^{\left(j\right)} & \equiv \overset{B}{\underset{b=1}{⊎}}\overset{n_{b}}{\underset{v=1}{⊎}}\left\{\Delta D_{b,v}^{\left(j\right)}\right\},\\ G_{\Delta ,\mathrm{cohort}}^{\left(j\right)} & \equiv \overset{B}{\underset{b=1}{⊎}}\overset{n_{b}}{\underset{v=1}{⊎}}\left\{\Delta G_{b,v}^{\left(j\right)}\right\}, \end{array}$$

with repeated values retained.

#### Per‐trial deltas

For each localization‐perturbation trial $t=1,\cdots ,N_{T}$,

(B19)
$$\Delta D_{b,v,t}\equiv D_{b,v}^{\left(0\right)}-D_{b,v}^{\left(t\right)},\;\Delta G_{b,v,t}\equiv G_{b,v}^{\left(0\right)}-G_{b,v}^{\left(t\right)}.$$

Cohort‐level statistics for per‐trial deltas were obtained by pooling across all biopsies, voxels, and trials as

(B20)
$$\begin{array}{cc} D_{\Delta } & \equiv \overset{B}{\underset{b=1}{⊎}}\overset{n_{b}}{\underset{v=1}{⊎}}{\left\{\Delta D_{b,v,t}\right\}}_{t=1}^{N_{T}},\\ G_{\Delta } & \equiv \overset{B}{\underset{b=1}{⊎}}\overset{n_{b}}{\underset{v=1}{⊎}}{\left\{\Delta G_{b,v,t}\right\}}_{t=1}^{N_{T}}. \end{array}$$

#### Directionality summary (CLES)

When pooling signed deltas, directionality was summarized using the common‐language effect size:

(B21)
$$\mathrm{CLES}=P\left(\Delta >0\right)+\frac{1}{2}P\left(\Delta =0\right),$$

where probabilities are taken with respect to the empirical distribution of deltas in the pooled sets above.

### B.6 DVH‐style metrics and QA rules

Let $D_{\mathrm{Rx}}=13.5\;\mathrm{Gy}$ denote the prescription per fraction. For each trial t, DVH‐style metrics were computed from the biopsy voxel doses ${\left\{D_{b,v}^{\left(t\right)}\right\}}_{v=1}^{n_{b}}$.

For x∈(0,100), we define

(B22)
$$D_{b,x\%}\left(t\right)\equiv Q_{1-x/100}\;\left({\left\{D_{b,v}^{\left(t\right)}\right\}}_{v=1}^{n_{b}}\right),$$

the minimum dose to the hottest x% of voxels, where $Q_{q}\left(\cdot \right)$ denotes the sample quantile.

For x∈(0,300),

(B23)
$$V_{b,x\%}\left(t\right)\equiv \frac{1}{n_{b}}\sum_{v=1}^{n_{b}}I\;\left[D_{b,v}^{\left(t\right)}\geq \left(\frac{x}{100}\right)D_{\mathrm{Rx}}\right],$$

is the fraction of biopsy voxels receiving at least $\left(x/100\right)D_{\mathrm{Rx}}$.

The four QA rules used in the main text are

(B24)
$$\begin{array}{cc} r=1 & :\;D_{b,2\%}\left(t\right)\geq 32\;\text{Gy},\\ r=2 & :\;D_{b,50\%}\left(t\right)\geq 27\;\text{Gy},\\ r=3 & :\;D_{b,98\%}\left(t\right)\geq 20\;\text{Gy},\\ r=4 & :\;V_{b,150\%}\left(t\right)\geq 50\%. \end{array}$$

For rule r we write $M_{b,r}\left(t\right)$ for the corresponding metric and $T_{r}$ for its threshold. The per‐trial pass indicator is

(B25)
$$I_{b,r}^{\left(t\right)}\equiv I\;\left[M_{b,r}\left(t\right)\geq T_{r}\right],\;t=0,1,\cdots ,N_{T},$$

with t=0 denoting the nominal (unshifted) configuration.

### B.7 Threshold pass probability and logistic models

#### Pass probability and margin

Threshold robustness under localization uncertainty was summarized by the Monte Carlo pass probability

(B26)
$$p_{b,r}\equiv \frac{1}{N_{T}}\sum_{t=1}^{N_{T}}I_{b,r}^{\left(t\right)},$$

computed over perturbed trials (t≥1). Biopsy‐rule pairs were classified as confident pass ($p_{b,r}\geq 0.95$), confident fail ($p_{b,r}\leq 0.05$), or borderline ($0.05<p_{b,r}<0.95$).

The signed nominal margin from threshold was defined as

(B27)
$$\delta_{b,r}\equiv M_{b,r}\left(0\right)-T_{r},$$

so that $\delta_{b,r}>0$ corresponds to a nominal pass and $\delta_{b,r}<0$ to a nominal fail.

#### Margin‐only logistic model

To relate $p_{b,r}$ to nominal predictors, the number of passing perturbed trials

(B28)
$$n_{\mathrm{pass},b,r}\equiv \sum_{t=1}^{N_{T}}I_{b,r}^{\left(t\right)}$$

was modeled as

(B29)
$$\begin{array}{cc} n_{\mathrm{pass},b,r} & ∼\mathrm{Binomial}\;\left(N_{T},\;\pi_{b,r}\right), \end{array}$$

(B30)
$$\begin{array}{cc} \mathrm{logit}(\pi_{b,r}) & =\beta_{0,r}+\beta_{1,r}\;\delta_{b,r}, \end{array}$$

fit separately for each rule r by maximum likelihood.

#### Margin plus secondary predictor

In the extended models, rule‐specific models were enriched with an additional biopsy‐level predictor value $g_{b,r}$ for biopsy b and rule r,

(B31)
$$\mathrm{logit}\left(\pi_{b,r}\right)=\beta_{0,r}+\beta_{1,r}\;\delta_{b,r}+\beta_{2,r}\;g_{b,r},$$

and nested likelihood‐ratio tests and ΔAIC were used to assess whether including $g_{b,r}$ improved fit.

#### Change in required margin and noise scale

For each rule r, threshold $T_{r}$, and its best secondary predictor ${\tilde{g}}_{r}$, we evaluated the fitted two‐predictor model (B31) at the 10th and 90th cohort percentiles of ${\tilde{g}}_{r}$ to obtain the corresponding margins ${\hat{\delta }}_{0.95,\mathrm{low}}$ and ${\hat{\delta }}_{0.95,\mathrm{high}}$. The effect was summarized as

(B32)
$$\Delta {\hat{\delta }}_{0.95}\equiv {\hat{\delta }}_{0.95,\mathrm{high}}-{\hat{\delta }}_{0.95,\mathrm{low}}.$$

To provide a scale‐free measure, $\Delta {\hat{\delta }}_{0.95}$ was normalized by a cohort‐level noise scale $\sigma_{r}$ defined as

(B33)
$$\sigma_{r}\equiv {\mathrm{median}}_{b}\left\{{\mathrm{SD}}_{t}\left(M_{b,r}\left(t\right)\right)\right\},$$

that is, the median across biopsies of the Monte Carlo standard deviation of the DVH metric $M_{b,r}\left(t\right)$ over trials.

### B.8 Voxel‐pair differences and length‐scale analysis

#### Pairwise difference matrices

For biopsy b, voxels $i,j\in \{1,\cdots ,n_{b}\}$, and trial t,

(B34)
$$M_{b,ij}^{D,(t)}=D_{b,i}^{\left(t\right)}-D_{b,j}^{\left(t\right)},\;M_{b,ij}^{G,(t)}=G_{b,i}^{\left(t\right)}-G_{b,j}^{\left(t\right)}.$$

By construction, $M_{b,ij}^{\cdot ,(t)}=-M_{b,ji}^{\cdot ,(t)}$ and $M_{b,ii}^{\cdot ,(t)}=0$.

#### Length‐scale absolute differences

Let the axial voxel spacing be Δz=1mm and define $\ell_{k}\equiv k\;\Delta z$. For a fixed separation k≥1,

(B35)
$$\begin{array}{cc} S_{b}^{D}\left(\ell_{k}\right) & \equiv \left\{\left|M_{b,ij}^{D,(t)}\right|:\;i<j,\;j-i=k,\;t=0,\cdots ,N_{T}\right\},\\ S_{b}^{G}\left(\ell_{k}\right) & \equiv \left\{\left|M_{b,ij}^{G,(t)}\right|:\;i<j,\;j-i=k,\;t=0,\cdots ,N_{T}\right\}. \end{array}$$

Cohort‐pooled sets were formed at each $\ell_{k}$ by pooling across biopsies,

(B36)
$$S_{\mathrm{cohort}}^{D}\left(\ell_{k}\right)\equiv \overset{B}{\underset{b=1}{⋃}}S_{b}^{D}\left(\ell_{k}\right),\;S_{\mathrm{cohort}}^{G}\left(\ell_{k}\right)\equiv \overset{B}{\underset{b=1}{⋃}}S_{b}^{G}\left(\ell_{k}\right),$$

with summary statistics (mean, median, IQR, and tail percentiles) computed per $\ell_{k}$. The number of contributing voxel pairs decreases at large $\ell_{k}$ because fewer biopsies satisfy $n_{b}\geq k+1$.

## APPENDIX C ADDITIONAL TABLES AND FIGURES

C8, C9, C10

**TABLE C1:** Summary statistics of dose and dose‐gradient values sampled across all uncertainty trials and biopsy voxels for the entire cohort, including the KDE‐based mode. These values correspond directly to the histograms shown in Figure 2.

Metric	Mode	Mean	Q50	SD	Min	Q05	Q25	Q75	Q95	Max
Dose (Gy)	17.7	27.2	22.0	19.6	8.2	15.5	18.1	28.5	54.4	663.0
Dose gradient ($\text{Gy}\;{\text{mm}}^{-1}$)	1.0	7.8	2.4	16.0	0.1	0.4	1.0	7.1	37.4	326.2

**TABLE C2:** Cohort‐level mean ± standard deviation of **voxel‐level** dosimetric statistics. For each voxel, statistics are first computed from the distribution across all uncertainty trials and then summarized across the 429 sampled biopsy voxels. The reported standard deviations therefore reflect inter‐voxel heterogeneity in these voxel‐level summaries.

	Central tendency and spread					
Metric	Nominal	Mean	Q50	SD	IQR	IPR90
Dose (Gy)	28.5 ± 18.8	27.2 ± 11.6	24.3 ± 8.1	9.8 ± 12.4	7.0 ± 7.7	28.1 ± 38.0
Dose gradient ($\text{Gy}\;{\text{mm}}^{-1}$)	10.7 ± 23.3	7.8 ± 10.1	5.0 ± 6.5	7.5 ± 10.0	8.4 ± 14.7	21.2 ± 29.3

	Extremes and quantiles					
Metric	Min	Q05	Q25	Q75	Q95	Max
Dose (Gy)	15.3 ± 2.8	19.3 ± 4.1	21.7 ± 5.8	28.7 ± 12.6	47.4 ± 40.2	112.7 ± 98.2
Dose gradient ($\text{Gy}\;{\text{mm}}^{-1}$)	0.5 ± 0.4	1.5 ± 1.3	2.7 ± 2.9	11.1 ± 17.1	22.6 ± 30.2	53.4 ± 54.0

**TABLE C3:** Cohort‐level mean ± standard deviation of **biopsy‐level** dosimetric statistics. For each biopsy, statistics are first computed from the distribution across all intra‐biopsy voxels and uncertainty trials and then summarized across the 27 sampled biopsies. The reported standard deviations therefore reflect inter‐biopsy heterogeneity in these biopsy‐level summaries.

	Central tendency and spread					
Metric	Nominal	Mean	Q50	SD	IQR	IPR90
Dose (Gy)	29.0 ± 15.2	27.6 ± 10.4	24.3 ± 7.5	12.4 ± 12.4	8.6 ± 8.0	31.8 ± 32.0
Dose gradient ($\text{Gy}\;{\text{mm}}^{-1}$)	11.5 ± 19.0	8.2 ± 8.7	4.7 ± 6.1	9.5 ± 10.7	7.5 ± 9.8	28.1 ± 34.4

	Extremes and quantiles					
Metric	Min	Q05	Q25	Q75	Q95	Max
Dose (Gy)	12.6 ± 2.2	17.9 ± 3.2	20.9 ± 4.9	29.5 ± 12.1	49.6 ± 33.7	173.9 ± 135.2
Dose gradient ($\text{Gy}\;{\text{mm}}^{-1}$)	0.4 ± 0.3	1.3 ± 1.2	2.5 ± 2.8	10.0 ± 12.5	29.4 ± 35.0	87.7 ± 72.2

**TABLE C4:** Cohort‐level summary of biopsy DVH metrics (N=27). For each biopsy and each DVH metric, Monte Carlo trial distributions were summarized by the statistics shown in each column. Entries are cohort mean ± standard deviation of those biopsy‐level statistics, so the reported standard deviations reflect inter‐biopsy heterogeneity.

	Central tendency and spread					
Metric	Nominal	Mean	Q50	SD	IQR	IPR90
$D_{2\%}$ (Gy)	41.6 ± 39.7	37.1 ± 18.2	29.7 ± 9.7	20.1 ± 24.2	14.4 ± 16.9	61.8 ± 78.4
$D_{50\%}$ (Gy)	28.2 ± 14.3	27.2 ± 10.7	24.8 ± 8.0	7.9 ± 8.8	6.9 ± 7.9	24.3 ± 29.9
$D_{98\%}$ (Gy)	22.1 ± 10.0	21.5 ± 7.9	20.4 ± 5.8	4.4 ± 6.4	4.5 ± 5.8	14.0 ± 21.1
$V_{100\%}$ (%)	99.8 ± 1.1	99.3 ± 2.4	99.6 ± 2.3	1.6 ± 2.5	0.4 ± 2.3	2.6 ± 7.0
$V_{125\%}$ (%)	90.8 ± 20.1	87.5 ± 20.1	89.8 ± 21.8	10.0 ± 8.5	9.0 ± 12.7	27.1 ± 27.8
$V_{150\%}$ (%)	66.1 ± 38.3	61.2 ± 35.0	63.0 ± 37.8	14.8 ± 10.0	17.4 ± 21.5	39.1 ± 32.3
$V_{175\%}$ (%)	48.7 ± 39.2	42.1 ± 33.2	42.1 ± 38.5	20.5 ± 12.0	25.6 ± 27.3	58.8 ± 37.3
$V_{200\%}$ (%)	31.7 ± 38.4	29.7 ± 29.6	28.0 ± 37.1	19.9 ± 12.5	22.9 ± 25.6	57.8 ± 38.6
$V_{300\%}$ (%)	14.9 ± 28.3	11.4 ± 16.7	6.3 ± 19.3	14.6 ± 13.9	17.5 ± 30.9	35.6 ± 36.3

	Extremes and quantiles					
Metric	Min	Q05	Q25	Q75	Q95	Max
$D_{2\%}$ (Gy)	17.3 ± 2.6	21.9 ± 4.2	25.4 ± 6.5	39.8 ± 21.3	83.7 ± 79.9	173.9 ± 135.2
$D_{50\%}$ (Gy)	15.8 ± 2.7	19.7 ± 3.8	22.2 ± 5.6	29.2 ± 12.7	44.0 ± 32.2	80.2 ± 48.6
$D_{98\%}$ (Gy)	13.1 ± 2.2	16.7 ± 3.0	18.6 ± 4.0	23.1 ± 9.4	30.7 ± 23.1	50.0 ± 39.8
$V_{100\%}$ (%)	70.9 ± 32.2	97.4 ± 7.0	99.3 ± 3.4	99.8 ± 1.1	100.0 ± 0.0	100.0 ± 0.0
$V_{125\%}$ (%)	29.2 ± 33.5	69.8 ± 31.4	84.3 ± 23.8	93.3 ± 20.2	96.8 ± 14.2	100.0 ± 0.0
$V_{150\%}$ (%)	4.1 ± 13.6	38.4 ± 38.1	53.3 ± 38.8	70.6 ± 37.3	77.5 ± 32.6	98.6 ± 6.1
$V_{175\%}$ (%)	1.9 ± 10.1	11.4 ± 25.4	30.2 ± 37.8	55.8 ± 39.7	70.2 ± 37.6	91.0 ± 18.3
$V_{200\%}$ (%)	0.0 ± 0.0	2.5 ± 13.2	18.0 ± 31.2	40.9 ± 38.6	60.4 ± 39.0	86.8 ± 22.1
$V_{300\%}$ (%)	0.0 ± 0.0	0.0 ± 0.0	1.8 ± 9.1	19.3 ± 32.7	35.6 ± 36.3	71.9 ± 31.2

**TABLE C5:** Cohort‐level summaries of voxelwise statistical‐summary deltas $\Delta D_{b,v}^{\left(j\right)}$ and $\Delta G_{b,v}^{\left(j\right)}$ and their absolute values, with $\Delta D_{b,v}^{\left(j\right)}$ and $\Delta G_{b,v}^{\left(j\right)}$ defined in Equation (B17) for j∈{mode,Q50,mean}. Medians, means, standard deviations (SD), interquartile ranges (IQR), and 5th/95th percentiles (Q05/Q95) are reported for both dose (Gy) and normed dose gradient ($\text{Gy}\;{\text{mm}}^{-1}$), pooled across all biopsies ($n_{\mathrm{biopsy}}=27$) and voxels ($n_{\mathrm{voxel}}=429$).

	Absolute dose deltas $|\Delta D_{b,v}^{\left(j\right)}|$ (Gy)						Absolute gradient deltas $|\Delta G_{b,v}^{\left(j\right)}|$ ($\text{Gy}\;{\text{mm}}^{-1}$)					
MC summary j	Median	Mean	SD	IQR	Q05	Q95	Median	Mean	SD	IQR	Q05	Q95
mode	1.2	6.0	15.2	5.0	0.0	28.7	0.7	8.1	21.3	5.5	0.0	44.1
Q50	0.9	4.6	13.7	3.3	0.0	22.2	0.5	6.1	18.7	2.7	0.0	32.6
mean	1.1	3.7	10.4	2.8	0.0	13.3	1.1	5.3	14.7	2.7	0.0	25.0

	Signed dose deltas $\Delta D_{b,v}^{\left(j\right)}$ (Gy)						Signed gradient deltas $\Delta G_{b,v}^{\left(j\right)}$ ($\text{Gy}\;{\text{mm}}^{-1}$)					
MC summary j	Median	Mean	SD	IQR	Q05	Q95	Median	Mean	SD	IQR	Q05	Q95
mode	1.0	5.7	15.3	5.2	−0.8	28.7	0.6	8.0	21.4	5.6	−0.3	44.1
Q50	0.4	4.1	13.9	3.2	−1.5	22.2	0.1	5.7	18.9	2.9	−1.0	32.6
mean	−0.2	1.2	11.0	2.1	−6.2	13.3	−0.3	2.9	15.4	1.9	−6.1	25.0

**TABLE C6:** Cohort‐level summary of nominal‐trial voxelwise differences $\Delta D_{b,v,t}$ and $\Delta G_{b,v,t}$ and their absolute values $|\Delta D_{b,v,t}|$ and $|\Delta G_{b,v,t}|$, as defined in Equations (B19) and (B20), pooled over all biopsies ($n_{\mathrm{biopsy}}=27$), voxels ($n_{\mathrm{voxel}}=429$), and trials ($N_{T}=10,000$ per voxel). IPR90 denotes the central 90% interval width, $Q_{95}-Q_{05}$.

	Absolute $|\Delta D_{b,v,t}|$, $|\Delta G_{b,v,t}|$								
Metric	Mean	SD	Median	IQR	IPR90	Min	Q05	Q95	Max
$|\Delta D_{b,v,t}|$ (Gy)	7.6	17.7	2.1	6.0	32.9	0.0	0.1	33.0	596.5
$|\Delta G_{b,v,t}|$ ($\text{Gy}\;{\text{mm}}^{-1}$)	7.7	18.5	1.3	6.2	37.2	0.0	0.0	37.2	221.4

	Signed $\Delta D_{b,v,t}$, $\Delta G_{b,v,t}$								
Metric	Mean	SD	Median	IQR	IPR90	Min	Q05	Q95	Max
$\Delta D_{b,v,t}$ (Gy)	1.2	19.2	0.4	4.3	34.7	−596.5	−15.0	19.7	202.3
$\Delta G_{b,v,t}$ ($\text{Gy}\;{\text{mm}}^{-1}$)	2.9	19.8	0.1	2.7	43.0	−221.4	−13.4	29.6	179.8

**TABLE C7:** Pearson correlations between voxel‐level dose deltas for the three Monte Carlo summary definitions $\Delta D_{b,v}^{\left(\mathrm{mode}\right)}$, $\Delta D_{b,v}^{\left(Q50\right)}$, and $\Delta D_{b,v}^{\left(\mathrm{mean}\right)}$, defined in (B17). Each correlation uses one row per voxel (429 voxels; N=27 biopsies), comparing either the signed deltas (top row) or their absolute values (bottom row). All two‐sided p‐values were $<10^{-190}$ (not shown); in all cases r≥0.94, indicating that the three delta definitions are highly collinear at the voxel level.

	$\Delta D_{b,v}^{\left(\mathrm{mode}\right)}$ vs. $\Delta D_{b,v}^{\left(Q50\right)}$		$\Delta D_{b,v}^{\left(Q50\right)}$ vs. $\Delta D_{b,v}^{\left(\mathrm{mean}\right)}$		$\Delta D_{b,v}^{\left(\mathrm{mode}\right)}$ vs. $\Delta D_{b,v}^{\left(\mathrm{mean}\right)}$	
Delta definition	n	r	n	r	n	r
Signed $\Delta D_{b,v}$	429	0.99	429	0.97	429	0.94
$|\Delta D_{b,v}|$	429	0.99	429	0.97	429	0.95

**TABLE C8:** Top ten per‐voxel‐defined dosimetric, geometric, and radiomic predictors ranked by Pearson correlation with voxel‐wise absolute dose deltas $|\Delta D_{b,v}^{\left(\mathrm{mode}\right)}|$. Correlations are computed over all voxels and biopsies in the cohort. Predictor notation follows Appendix A.

Predictor	r (Pearson)	ρ (Spearman)
**Mode**: voxel‐wise absolute dose deltas $|\Delta D_{b,v}^{\left(\mathrm{mode}\right)}|$		
Nominal voxel dose $D_{b,v}^{\left(0\right)}$ (Gy)	0.95	0.79
Nominal voxel dose gradient $G_{b,v}^{\left(0\right)}$ ($\text{Gy}\;{\text{mm}}^{-1}$)	0.83	0.87
Normalized biopsy‐prostate centroid distance ${\bar{d}}_{P,v}^{\mathrm{norm},\mathrm{cen}}$ (dimensionless)	0.30	0.38
DIL flatness ${\mathrm{Flatness}}_{\mathrm{DIL}}$ (dimensionless)	0.27	0.04
Prostate left‐right dimension at centroid $d_{\mathrm{LR},\mathrm{prostate}}$ (mm)	−0.26	−0.12
Normalized DIL‐prostate centroid distance $d_{\mathrm{DIL},\mathrm{prostate}}^{\mathrm{norm}}$ (dimensionless)	0.25	0.37
DIL major axis (equivalent ellipsoid) $a_{\mathrm{DIL},\mathrm{maj}}$ (mm)	−0.24	−0.20
Core length $L_{b}$ (mm)	−0.24	−0.36
DIL maximum 3D diameter $D_{\mathrm{DIL}}^{\max}$ (mm)	−0.24	−0.22
DIL PCA major axis $\lambda_{\mathrm{DIL},\mathrm{major}}^{\mathrm{PCA}}$ (mm)	−0.23	−0.20

**TABLE C9:** Top ten per‐voxel‐defined dosimetric, geometric, and radiomic predictors ranked by Pearson correlation with voxel‐wise absolute dose deltas $|\Delta D_{b,v}^{\left(\mathrm{median}\right)}|$. Correlations are computed over all voxels and biopsies in the cohort. Predictor notation follows Appendix A.

Predictor	r (Pearson)	ρ (Spearman)
**Median**: voxel‐wise absolute dose deltas $|\Delta D_{b,v}^{\left(\mathrm{median}\right)}|$		
Nominal voxel dose $D_{b,v}^{\left(0\right)}$ (Gy)	0.91	0.74
Nominal voxel dose gradient $G_{b,v}^{\left(0\right)}$ ($\text{Gy}\;{\text{mm}}^{-1}$)	0.77	0.76
Normalized biopsy‐prostate centroid distance ${\bar{d}}_{P,v}^{\mathrm{norm},\mathrm{cen}}$ (dimensionless)	0.25	0.33
Prostate surface‐area‐to‐volume ratio $A_{P}/V_{P}$ (${\mathrm{mm}}^{-1}$)	0.22	0.36
Core length $L_{b}$ (mm)	−0.21	−0.47
Prostate left‐right dimension at centroid $d_{\mathrm{LR},\mathrm{prostate}}$ (mm)	−0.20	−0.31
DIL flatness ${\mathrm{Flatness}}_{\mathrm{DIL}}$ (dimensionless)	0.20	0.04
DIL minor axis (equivalent ellipsoid) $a_{\mathrm{DIL},\min}$ (mm)	−0.20	−0.13
Prostate mean dimension at centroid ${\bar{d}}_{\mathrm{prostate}}$ (mm)	−0.19	−0.29
Normalized DIL‐prostate centroid distance $d_{\mathrm{DIL},\mathrm{prostate}}^{\mathrm{norm}}$ (dimensionless)	0.19	0.21

**TABLE C10:** Top ten per‐voxel‐defined dosimetric, geometric, and radiomic predictors ranked by Pearson correlation with voxel‐wise absolute dose deltas $|\Delta D_{b,v}^{\left(\mathrm{mean}\right)}|$. Correlations are computed over all voxels and biopsies in the cohort. Predictor notation follows Appendix A.

Predictor	r (Pearson)	ρ (Spearman)
**Mean**: voxel‐wise absolute dose deltas $|\Delta D_{b,v}^{\left(\mathrm{mean}\right)}|$		
Nominal voxel dose $D_{b,v}^{\left(0\right)}$ (Gy)	0.89	0.68
Nominal voxel dose gradient $G_{b,v}^{\left(0\right)}$ ($\text{Gy}\;{\text{mm}}^{-1}$)	0.73	0.71
Prostate left‐right dimension at centroid $d_{\mathrm{LR},\mathrm{prostate}}$ (mm)	−0.23	−0.31
Core length $L_{b}$ (mm)	−0.22	−0.47
Normalized biopsy‐prostate centroid distance ${\bar{d}}_{P,v}^{\mathrm{norm},\mathrm{cen}}$ (dimensionless)	0.21	0.22
Prostate surface‐area‐to‐volume ratio $A_{P}/V_{P}$ (${\mathrm{mm}}^{-1}$)	0.20	0.34
DIL flatness ${\mathrm{Flatness}}_{\mathrm{DIL}}$ (dimensionless)	0.20	0.05
Prostate mean dimension at centroid ${\bar{d}}_{\mathrm{prostate}}$ (mm)	−0.20	−0.30
DIL minor axis (equivalent ellipsoid) $a_{\mathrm{DIL},\min}$ (mm)	−0.19	−0.15
DIL major axis (equivalent ellipsoid) $a_{\mathrm{DIL},\mathrm{maj}}$ (mm)	−0.19	−0.16

**TABLE C11:** Cohort‐level Monte Carlo threshold QA summary (27 biopsies). Here $p_{b}$ denotes the pass probability $p_{b,r}$ for a given biopsy and DVH threshold rule. Borderline denotes $0.05<p_{b}<0.95$. The median margin and quartiles are computed from the nominal distance‐from‐threshold values $\delta_{b,r}$. ${\hat{\delta }}_{0.95}$ is the logit‐estimated nominal margin at which $p_{b}=0.95$ under the single‐predictor (margin‐only) model. Nominal misclassification (disagreement between nominal pass/fail and confident Monte Carlo classification) was zero for all thresholds and is therefore not tabulated. For the $D_{2\%}\geq 32\;\mathrm{Gy}$ threshold, one biopsy with extreme leverage on the margin‐only logistic fit was treated as an outlier and excluded from the model‐based summary, so counts for this row sum to 26.

Threshold	Median $p_{b}$ (IQR)	Conf. pass	Borderline	Conf. fail	Median margin ($Q_{25}$, $Q_{75}$)	${\hat{\delta }}_{0.95}$
$D_{2\%}\geq 32\;\mathrm{Gy}$	0.26 (0.08‐0.63)	1/26 (4%)	19/26 (73%)	6/26 (23%)	−4.6Gy (−9.4, 9.3Gy)	48.9Gy
$D_{50\%}\geq 27\;\mathrm{Gy}$	0.19 (0.01‐0.58)	1/27 (4%)	17/27 (63%)	9/27 (33%)	−2.4Gy (−7.1, 3.2Gy)	20.8Gy
$D_{98\%}\geq 20\;\mathrm{Gy}$	0.25 (0.01‐0.93)	4/27 (15%)	12/27 (44%)	11/27 (41%)	−1.2Gy (−3.0, 3.0Gy)	6.3Gy
$V_{150\%}\geq 50\%$	0.72 (0.31‐0.99)	10/27 (37%)	12/27 (44%)	5/27 (19%)	28.6% (−7.4%, 50.0%)	48.9%

**TABLE C12:** Logistic regression parameters for the margin‐only models fitted to Monte Carlo pass probabilities. The model is $\mathrm{logit}\left(\pi_{b,r}\right)=\beta_{0}+\beta_{\delta }\;\delta_{b,r}$, where $\pi_{b,r}$ is the underlying pass probability for biopsy b and rule r, and $\delta_{b,r}$ is the nominal distance‐from‐threshold expressed in the same units as the corresponding DVH metric (Gy for $D_{2\%}$, $D_{50\%}$, and $D_{98\%}$, percentage points for $V_{150\%}$). ${\hat{\delta }}_{0.50}$ and ${\hat{\delta }}_{0.95}$ denote the nominal margins at which the fitted model predicts $\pi_{b,r}=0.5$ and $\pi_{b,r}=0.95$, respectively. Weighted RMSE is computed at the biopsy level using the Monte Carlo weights, and McFadden's $R^{2}$ quantifies overall model fit relative to an intercept‐only model.

Threshold	N	$\beta_{0}$	$\beta_{\delta }$ ^1^	OR / 1 unit^2^	OR / 5 units^2^	${\hat{\delta }}_{0.50}$	${\hat{\delta }}_{0.95}$	wRMSE($p_{b}$)	McFadden $R^{2}$
$D_{2\%}\geq 32\;\mathrm{Gy}$	26	−0.93	0.08	1.08	1.49	11.7	48.9	0.15	0.29
$D_{50\%}\geq 27\;\mathrm{Gy}$	27	−1.02	0.19	1.21	2.60	5.4	20.8	0.12	0.44
$D_{98\%}\geq 20\;\mathrm{Gy}$	27	−0.72	0.58	1.78	18.0	1.3	6.3	0.15	0.57
$V_{150\%}\geq 50\%$	27	−0.42	0.07	1.07	1.41	6.2	48.9	0.11	0.60

^1^ Units of $\beta_{\delta }$ are the inverse of the margin units: ${\mathrm{Gy}}^{-1}$ for dose‐based thresholds and (percentage point) for $V_{150\%}$.

^2^ For $D_{2\%}$, $D_{50\%}$, and $D_{98\%}$, OR / 1 Gy and OR / 5 Gy denote the odds ratios for passing associated with 1 Gy and 5 Gy increases in nominal margin, respectively. For $V_{150\%}$, they correspond to 1 and 5 percentage points of margin.

**TABLE C13:** Nested logistic model comparison and effect of the best secondary predictor ${\tilde{g}}_{r}$ on the nominal margin required to achieve a Monte Carlo pass probability of $p_{b}=0.95$ for each DVH rule r. For each rule, the single‐predictor (margin‐only) model uses only the nominal distance‐from‐threshold $\delta_{b,r}$, and the two‐predictor model adds the best biopsy‐level candidate ${\tilde{g}}_{r}$. ΔAIC is defined as ${\mathrm{AIC}}_{\text{2D}}-{\mathrm{AIC}}_{\text{margin-only}}$, and “LR p” is the likelihood‐ratio test p‐value for including ${\tilde{g}}_{r}$. The secondary predictor is evaluated at the 10th and 90th cohort percentiles $q_{0.10}\left({\tilde{g}}_{r}\right)$ and $q_{0.90}\left({\tilde{g}}_{r}\right)$, with the corresponding model‐based 95%‐pass margins ${\hat{\delta }}_{0.95}$ reported (“Low” and “High”). Here ${\hat{\delta }}_{0.95}$ denotes the nominal distance from threshold $T_{r}$ at which the fitted logistic model predicts $p_{b}=0.95$. The difference $\Delta {\hat{\delta }}_{0.95}$ is the change in required margin when moving from the low to the high secondary‐predictor value (negative values indicate a reduced required margin). $\sigma_{r}$ is the cohort‐median biopsy‐level Monte Carlo standard deviation of the metric $M_{b,r}$, and the final column reports the dimensionless ratio $\Delta {\hat{\delta }}_{0.95}/\sigma_{r}$. Rectum nearest‐neighbor distance ${\bar{d}}_{R}^{\mathrm{NN}}$ denotes the biopsy‐level mean signed distance from sampled biopsy points to the rectal boundary representation, and ${\bar{G}}_{b}^{\left(0\right)}$ is the nominal core‐averaged dose gradient as defined in Equation (B10).

Rule r	Secondary predictor ${\tilde{g}}_{r}$	ΔAIC	LR p	$q_{0.10}\left({\tilde{g}}_{r}\right)$	$q_{0.90}\left({\tilde{g}}_{r}\right)$	Low ${\hat{\delta }}_{0.95}$	High ${\hat{\delta }}_{0.95}$	$\Delta {\hat{\delta }}_{0.95}$	$\sigma_{r}$	$\Delta {\hat{\delta }}_{0.95}/\sigma_{r}$
$D_{2\%}\geq 32\;\mathrm{Gy}$	${\bar{G}}_{b}^{\left(0\right)}$	+1.6	0.53	0.65 $\text{Gy}\;{\text{mm}}^{-1}$	16.8 $\text{Gy}\;{\text{mm}}^{-1}$	28.8 Gy	38.0 Gy	9.3 Gy	11.5 Gy	0.81
$D_{50\%}\geq 27\;\mathrm{Gy}$	${\mathrm{SphDisp}}_{\mathrm{DIL}}$	+1.4	0.46	1.19	1.50	21.8 Gy	16.7 Gy	−5.2 Gy	6.0 Gy	−0.85
$D_{98\%}\geq 20\;\mathrm{Gy}$	${\bar{G}}_{b}^{\left(0\right)}$	+1.3	0.41	0.66 $\text{Gy}\;{\text{mm}}^{-1}$	22.3 $\text{Gy}\;{\text{mm}}^{-1}$	5.4 Gy	6.3 Gy	0.9 Gy	2.4 Gy	0.37
$V_{150\%}\geq 50\%$	${\bar{d}}_{R}^{\mathrm{NN}}$ (mm)	+1.4	0.43	9.1 mm	24.2 mm	42.7%	61.2%	18.5%	12.0%	1.54
