# Correction to: “Probabilistic quality assurance of biopsy DVH thresholds in HDR prostate brachytherapy under localization uncertainty”

**DOI:** 10.1002/acm2.70852

**Published:** 2026-10-14

## Article being corrected

Muscat M, Crook J, Jirasek A, Andrews J, Becker N. Probabilistic quality assurance of biopsy DVH thresholds in HDR prostate brachytherapy under localization uncertainty. *J Appl Clin Med Phys*. 2026;27:e70660. https://doi.org/10.1002/acm2.70660

A bibliographic correction is required for Reference 31. At the time of submission of the original article, the referenced companion manuscript had not yet been published and was therefore cited using its pre‐publication title and status. The article has since been published with a revised title and final publication details.

### Current reference 31

Muscat M, Crook J, Jirasek A, Andrews J, Becker N. Millimetre‐scale dosimetry along MR‐US guided prostate core‐needle biopsies in HDR brachytherapy with explicit modelling of localization uncertainty. *Phys Med Biol*. 2025. Submitted.

### Corrected reference 31

Muscat M, Crook J, Jirasek A, Andrews J, Becker N. Dosimetric analysis along core‐needle biop‐sies in prostate HDR brachytherapy. *Phys Med Biol*. 2026;71(16):165017. doi:10.1088/1361‐6560/ae9823.

No other changes to the article are required.

